# Plasma Lipid Composition and Risk of Developing Cardiovascular Disease

**DOI:** 10.1371/journal.pone.0071846

**Published:** 2013-08-15

**Authors:** Celine Fernandez, Marianne Sandin, Julio L. Sampaio, Peter Almgren, Krzysztof Narkiewicz, Michal Hoffmann, Thomas Hedner, Björn Wahlstrand, Kai Simons, Andrej Shevchenko, Peter James, Olle Melander

**Affiliations:** 1 Department of Clinical Sciences, Lund University, Malmö, Sweden; 2 Department of Immunotechnology, Lund University, Lund, Sweden; 3 Max Planck Institute of Molecular Cell Biology and Genetics, Dresden, Germany; 4 Department of Hypertension and Diabetology, Medical University of Gdansk, Gdansk, Poland; 5 Department of Medicine, Sahlgrenska Academy, Goteborg University, Goteborg, Sweden; Innsbruck Medical University, Austria

## Abstract

**Aims:**

We tested whether characteristic changes of the plasma lipidome in individuals with comparable total lipids level associate with future cardiovascular disease (CVD) outcome and whether 23 validated gene variants associated with coronary artery disease (CAD) affect CVD associated lipid species.

**Methods and Results:**

Screening of the fasted plasma lipidome was performed by top-down shotgun analysis and lipidome compositions compared between incident CVD cases (n = 211) and controls (n = 216) from the prospective population-based MDC study using logistic regression adjusting for Framingham risk factors. Associations with incident CVD were seen for eight lipid species (0.21≤q≤0.23). Each standard deviation unit higher baseline levels of two lysophosphatidylcholine species (LPC), LPC16∶0 and LPC20∶4, was associated with a decreased risk for CVD (*P = *0.024–0.028). Sphingomyelin (SM) 38∶2 was associated with increased odds of CVD (*P = *0.057). Five triglyceride (TAG) species were associated with protection (*P = *0.031–0.049). LPC16∶0 was negatively correlated with the carotid intima-media thickness (*P = *0.010) and with HbA1c (*P = *0.012) whereas SM38∶2 was positively correlated with LDL-cholesterol (*P* = 0.0*10^−6^) and the q-values were good (q≤0.03). The risk allele of 8 CAD-associated gene variants showed significant association with the plasma level of several lipid species. However, the q-values were high for many of the associations (0.015≤q≤0.75). Risk allele carriers of 3 CAD-loci had reduced level of LPC16∶0 and/or LPC 20∶4 (*P*≤0.056).

**Conclusion:**

Our study suggests that CVD development is preceded by reduced levels of LPC16∶0, LPC20∶4 and some specific TAG species and by increased levels of SM38∶2. It also indicates that certain lipid species are intermediate phenotypes between genetic susceptibility and overt CVD. But it is a preliminary study that awaits replication in a larger population because statistical significance was lost for the associations between lipid species and future cardiovascular events when correcting for multiple testing.

## Introduction

Cardiovascular mortality and morbidity is a major public health problem in Western societies. Traditional cardiovascular risk factors do not fully explain future cardiovascular events [1], [2] and adding modern biomarkers to the standard risk factors has, thus so far, only proven to minimally improve individual risk prediction [3], [4], thus underlining the need to identify new biomarkers.

Lipids are thought to play a central role in cardiovascular disease (CVD) development and total plasma triglycerides and cholesterol as well as LDL- and HDL-cholesterol are traditionally monitored as predictors of cardiovascular events. However, those are crude measurements of the sum of a complex composition of lipids and do not at all reflect other potentially atherogenic lipid species. We here hypothesized that specific plasma lipid species, rather than the rough phenotype of total triglycerides and cholesterol may be altered in subjects who develop CVD later in life, implying that they may be involved in the CVD pathogenesis.

Lipidomics, a subset within the field of metabolomics, strives to quantitatively describe the complete set of all lipids in a given cell type, tissue or biologic fluid of interest at a given time [5]. There is no single instrument or approach that can currently do so, but instead multiple and often complementary analytical approaches can be employed. Typically, global lipid profiling is conducted by directly infusing a crude lipid extract into the mass spectrometer without prior chromatographic separation, also called shotgun technique [6], or by using on-line liquid chromatographic separation prior mass spectrometry (MS) analysis [7]. Lipidomic analyses for human biomarker discovery using either approach are now emerging [8]–[10].

Shotgun lipidomics which allows high-throughput, high inter-sample reproducibility, high sensitivity and ease of automation [11] was here used for screening of the plasma lipidome in a case-control material derived from a prospective population-based cohort study with similar plasma total lipids level. A top-down approach where individual lipid species are identified by accurately determining precursor masses with no recourse to tandem MS was implemented as previously described [8], [12].

Because the mechanisms underlying CVD for most of the reported CVD-associated gene variants are unknown, we also tested whether the plasma lipidome associates with 23 well-validated gene variants for risk of coronary artery disease [13].

## Materials and Methods

### Ethics Statement

The Malmö Diet and Cancer study was approved by the Ethics Committee at Lund University and all participants provided written informed consent.

### Study Participants and Data Collection

The Malmö Diet and Cancer (MDC) study is a population-based, prospective epidemiologic cohort consisting of 28,449 individuals who attended a baseline examination between 1991 and 1996 [14]. From the MDC cohort, 6,103 persons were randomly selected and asked to participate in a cardiovascular cohort (MDC-CC) between 1991 and 1994, which was designed to study the epidemiology of carotid artery disease [15], [16]. All participants underwent a medical history assessment, a physical examination and a laboratory assessment of cardiovascular risk factors, including blood pressure, presence of diabetes mellitus (ascertained from self-reporting, or use of anti-diabetic medication, or fasting whole blood glucose >6.1 mM), smoking status, antihypertensive medication, a fasted lipid profile, C-reactive protein (CRP) and measurement of the common carotid intima-media thickness (IMT) by ultrasound [16], [17]. 23 validated gene variants associated with coronary artery disease (CAD) [13], [18]–[20] were genotyped (Supplementary Table S6). Genotyping was performed using SEQUENOM MassARRAY® Designer software and oligonucleotides were provided by Metabion (Martinsried, Germany). Assays were performed on the SEQUENOM Maldi-Tof mass spectrometer (San Diego, CA) using iPLEX reagents and protocols and 10 ng DNA as PCR template.

During a mean follow-up time of 12.2±2.3 years [21], 364 first incident cardiovascular events (myocardial infarction, ischemic stroke and death from coronary heart disease) with complete baseline clinical information were ascertained from three registries: the Swedish Hospital Discharge Register, the Swedish Cause of Death Register and the Stroke Register of Malmö, as previously described [17]. We matched incident cardiovascular disease (CVD) cases with CVD free control subjects based on gender, age (±1 year) and Framingham risk score [22] (<0.1% difference in 10 year estimated risk) and also required that the follow-up time of the control was at least as long as that of the corresponding incident CVD case. These criteria resulted in successful matching of 253 CVD cases with 253 controls. Out of those, plasma was missing for 46 individuals. Moreover, 45 samples were lost after lipid extraction. This left 211 CVD cases and 216 controls for lipid profiling.

### Materials, Chemicals and Lipid Standards

Material resistant to organic solvent was used (e.g. polypropylene, silicone, Teflon). Synthetic lipid standards were purchased from Avanti Polar Lipids, Inc. (Alabaster, AL) or Larodan Fine Chemicals (Malmö, Sweden). Methyl-tert-butylether (MTBE) and water (LiChrosolv grade) were purchased from Merck (Darmstadt, Germany). Methanol, chloroform and ammonium acetate (Liquid Chromatography grade) were purchased from Fluka (Buchs SG, Switzerland) and 2-propanol (ACS grade) from Sigma-Aldrich (Munich, Germany).

### Lipid Extraction

Overnight fasted citrate samples placed at −80°C immediately after collection, between 1991 and 1994, were analyzed. The samples had never been previously thawed. The samples were randomized before the lipid extraction step, which was carried out successively for all the samples. Lipid extraction was performed as previously described [23] but with adjustments in order to automate the procedure. In brief, 5 µL of plasma were manually pipetted into a 96-well plate (deep well plate from Axygen Scientific with ImperaMat) placed on ice whereas the following pipetting steps were performed at room temperature in a liquid-handling station (Beckman BiomekFX) using ART filter tips and polypropylene reagent reservoirs (FluidX). Samples were spiked with 350 µL of internal standard lipid mixture in MTBE/methanol 5/1.5 (v/v) providing a total of 2.7 nmol cholesteryl heptadecanoate (CE17∶0), 0.7 nmol heptadecanoyl sphingomyelin (SM17∶0), 3.5 nmol 1,2-di-O-hexadecyl-*sn*-glycero-3-phosphocholine (PC-O12∶0/−O12∶0), 0.9 nmol 1,2-di-O-phytanyl-*sn*-glycero-3-phosphoethanolamine (PE-O16∶0/−O16∶0), 3.1 nmol 1-lauroyl-2-hydroxy-*sn*-glycero-3-phosphocholine (LPC12∶0), 0.4 nmol N-heptadecanoyl-D-*erythro*-sphingosine (Cer17∶0), 3.1 nmol trilaurin (TAG12∶0) and 0.5 nmol dilaurin (DAG12∶0). ). Then 350 µL of MTBE/methanol 5∶1.5 were added and the samples were shaken at 4°C for 1 h. Afterwards, 150 µL of water were added, followed by shaking at 4°C for 10 min and centrifugation for 5 min at 4,000 rpm on Rotanta 460R centrifuge (Hettich, Tuttlingen, Germany). The upper organic phase was transferred into a 96-well plate with glass inserts and a silicone/Teflon coated sealing mat (Chromacol) and stored at −20°C until performing the MS analysis for all the samples successively.

### Shotgun Screening of Plasma Lipidome

Prior to the MS analysis, the lipid extracts were diluted 10 times with a mixture of chloroform/methanol/2-propanol 1/2/4 (v/v/v) containing 7.5 mM ammonium acetate and placed in a 96-well plate (Eppendorf) that was then sealed with aluminium foil (Corning). Shotgun analysis was performed on a LTQ Orbitrap (Thermo Fisher Scientific, Waltham, MA) coupled to a TriVersa NanoMate robotic nanoflow ion source (Advion BioSciences, Ithaca, NY) [8], [12]. Samples were analyzed in duplicate. Lipids were identified and quantified using the LipidXplorer software [24] and lipid species of the following lipid classes were recognized: triacylglyceride (TAG), diacylglyceride (DAG), cholesteryl ester (Chol-FA), sphingomyelin (SM), phosphatidylcholine (PC), PC-ether (PC-O), lyso-PC (LPC), phosphatidylethanolamine (PE) and PE-ether (PE-O). Identification of the different lipid species was based on MS survey scans acquired in positive ion mode in the Orbitrap analyzer at a target mass resolution of 100,000 using a mass accuracy of better than 5 ppm and a signal to noise ratio of 2. Lipid species were quantified by normalizing the intensities of their peaks to the intensity of the peaks of internal standards spiked into the sample prior to lipid extraction. The internal standards were also used to monitor the quality of the MS analysis and representative mass spectra are presented (Supplementary Figure S1A and S1B). An internal standard mix was both extracted and run independently 18 times across the entire analysis to get an estimate of the coefficient of variation of the combined lipid extraction and MS analysis from the internal standards (Supplementary Table S1). The maximum value of duplicate samples was kept. Lipid species with >30% missing observations were excluded.

### Statistical Analyses

SPSS (version 18.0) was used for all statistical analyses. Data were assessed for normality with histograms. Due to non-normality all the lipid species were log transformed prior analysis. All tests were two-sided and data were considered significant if *P*<0.05.

To determine the association of baseline individual lipid species with future CVD, we performed binary logistic regression adjusting for age, sex, diabetes, smoking status, LDL-cholesterol, HDL-cholesterol, systolic blood pressure (SBP), body mass index (BMI) and use of anti-hypertensive treatment.

Q-values were calculated using the QVALUE software [25].

Hierarchical clustering was performed with Euclidean distance and average linkage in MATLAB R2011a (version 7.12.0.635).

## Results

### Lipid Metabolites Profiling in the Cardiovascular Cohort of the Malmö Diet and Cancer Study

As a result of the initial matching procedure (age, gender and Framingham risk score) the baseline characteristics of the 211 incident cases of CVD and 216 control subjects were similar for most risk factors except fasted plasma glucose level and diabetes. The frequency of use of lipid lowering drugs was low (Table 1).

**Table 1:** Baseline characteristics of the study samples.

Characteristic	Control (n = 216)	CVD case (n = 211)	*P* value
Age (years)	60.7±5.1	60.2±5.3	0.331
Women (%)	47.7	47.4	0.952
BMI (kg/m^2^)	26.3±4.3	26.5±4.4	0.568
Systolic blood pressure (mm Hg)	149.3±19.8	149.7±18.4	0.815
Diastolic blood pressure (mm Hg)	90.1±9.6	90.1±9.5	0.981
Glucose (mmol/l)	5.2±1.2	5.7±2.2	0.008
Cholesterol (mmol/l)	6.3±1.1	6.3±1.0	0.611
Triglycerides (mmol/l)	1.5±0.7	1.4±0.6	0.136
High density lipoprotein (mmol/l)	1.3±0.3	1.3±0.3	0.541
Low density lipoprotein (mmol/l)	4.3±1.0	4.4±1.0	0.426
Diabetes (%)	8.3	15.2	0.028
Current smoker (%)	33.3	33.6	0.945
Anti-hypertensive treatment (%)	25.9	23.7	0.594
Lipid lowering drugs (%)	0.9	3.8	0.050

Values are mean±s.d. or percentage. *P* values were calculated using a t test for continuous variables and Pearson Chi-Square for binary variables.

**Figure 1:**
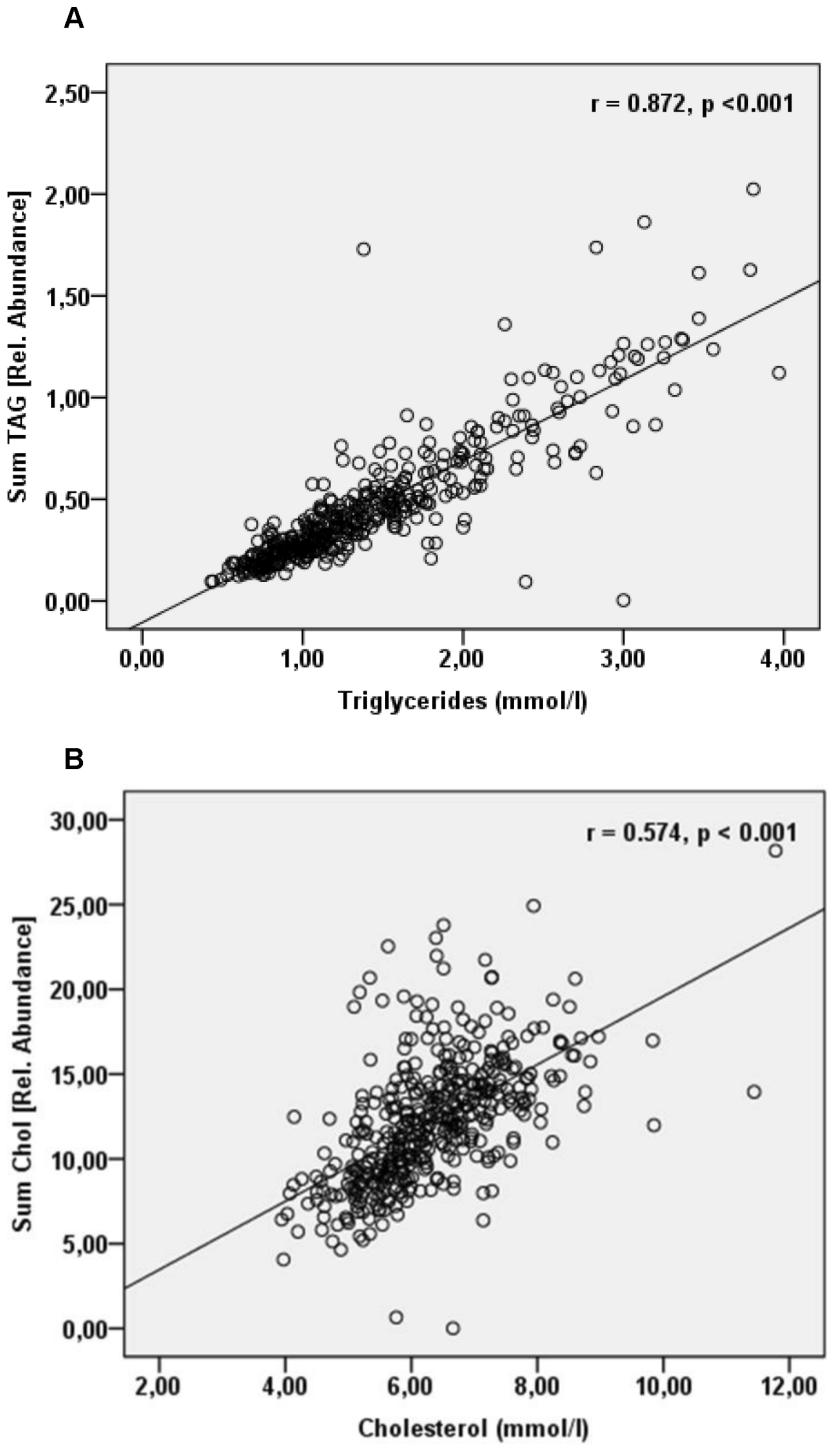
Quantification by top-down lipidomics correlates with clinical parameters. Linear regression analysis of A) the total triglyceride content or B) the total cholesterol content determined by MS versus the value obtained by traditional clinical chemistry analysis. The total triglyceride content measured by MS is obtained by summing the abundances of all the individual TAG species and the total cholesterol content by summing the abundances of free cholesterol and all cholesteryl esters.

Lipid profiling was performed on samples obtained from the baseline examination that took place between 1991 and 1994. A total of 85 lipid species belonging to 9 major lipid classes were identified and quantified by the approach used (Supplementary Table S2). The total quantities of triglycerides and cholesterol determined by mass spectrometry were correlated with the values obtained by traditional clinical chemistry analysis (Figure 1 and Supplementary Figure S2). As known from previous study, the correlation was substantially stronger for triglycerides than for cholesterol [8].

### Selected Lipid Species Associate with Future Adverse Cardiovascular Disease Outcome

Binary logistic regression was performed to assess the association between baseline lipid species level and future CVD adjusting for Framingham risk factors. Associations with incident CVD were seen for lipid species belonging to the lysophosphatidylcholine (LPC), sphingomyelin (SM) and triacylglyceride (TAG) lipid classes, but the q-values for the associations were rather high (0.21≤ q ≤0.23) (Tables 2, 3 and Supplementary material online, Table S3A). Similar results were obtained when only adjusting for diabetes (Supplementary Table S3B).

**Table 2:** Relation of baseline phospholipids level to future adverse cardiovascular outcome adjusting for Framingham risk factors.

Model	LPC16∶0 (n = 424)	LPC20∶4 (n = 353)	SM38∶2 (n = 318)
Models adjusting for sex, age, BMI, type 2 diabetes, anti-hypertension treatment, smoking, LDL, HDL and SBP			
Lipid specie as continuous variable			
Per s.d.	0.79 (0.65–0.97)	0.77 (0.61–0.96)	1.28 (0.99–1.64)
*P*	0.028	0.024	0.057
q-value	0.210	0.210	0.228
**Lipid specie as categorical variable**			
First quartile	1.0 (referent)	1.0 (referent)	1.0 (referent)
Second quartile	1.21 (0.69–2.11)	1.13 (0.61–2.07)	0.94 (0.49–1.81)
Third quartile	0.94 (0.54–1.65)	0.62 (0.34–1.16)	1.320 (0.68–2.56)
Fourth quartile	0.57 (0.32–1.00)	0.62 (0.33–1,17)	1.85 (0.92–3.71)
*P* for trend	0.032	0.048	0.054

Values are odds ratios (95% confidence intervals) for cardiovascular disease from multivariate adjusted binary logistic regressions performed with the Z score of a given lipid specie obtained after log transformation. BMI, body mass index; HDL, high-density lipoprotein cholesterol; LDL, low-density lipoprotein cholesterol; LPC, lysophosphatidylcholine; SBP, systolic blood pressure; SM, sphingomyelin.

**Table 3:** Relation of baseline triglycerides specie level to future adverse cardiovascular outcome adjusting for Framingham risk factors.

Model	TAG48∶1 (n = 424)	TAG48∶2 (n = 424)	TAG48∶3 (n = 402)	TAG50∶3 (n = 424)	TAG50∶4 (n = 423)
Models adjusting for sex, age, BMI, type 2 diabetes, anti-hypertension treatment, smoking, LDL, HDL and SBP					
Lipid specie as continuous variable					
Per s.d.	0.78 (0.63–0.98)	0.79 (0.64–0.98)	0.81 (0.65–1.00)	0.79 (0.63–0.98)	0.79 (0.64–0.98)
*P*	0.031	0.034	0.049	0.036	0.033
q-value	0.210	0.210	0.228	0.210	0.210

Values are odds ratios (95% confidence intervals) for cardiovascular disease from multivariate adjusted binary logistic regressions performed with the Z score of a given triacylglyceride specie obtained after log transformation. BMI, body mass index; HDL, high-density lipoprotein cholesterol; LDL, low-density lipoprotein cholesterol; SBP, systolic blood pressure; TAG, triacylglyceride.

In the LPC class, each standard deviation (SD) unit higher baseline levels of LPC16∶0 or LPC20∶4 was associated with a decreased risk of developing CVD over the 12-year follow-up period (OR = 0.79; *P = *0.028 and OR = 0.77; *P = *0.024, respectively) (Table 2). Individuals whose plasma level of LPC16∶0 or LPC20∶4 was in the top quartile had decreased odds of future CVD compared with individuals in the lowest quartile (OR = 0.57; *P* = 0.032 and OR = 0.62; *P* = 0.048, respectively) (Table 2).

SM38∶2, with a borderline *P*-value, was the only lipid specie of its class to be associated with increased odds of future CVD (OR = 1.28; *P* = 0.057). Individuals in the top quartile of baseline SM 38∶2 plasma level had an increased risk of developing CVD (OR = 1.28; *P* = 0.054) (Table 2).

In the TAG class, plasma levels of TAG48∶1, TAG48∶2, TAG48∶3, TAG50∶3 and TAG50∶4, were associated with decreased odds of future CVD (OR = 0.78–0.81; *P = *0.031–0.049) (Table 3). However, the quartiles analysis showed poor linearity between the various TAGs and CVD risk (Supplementary material online, Table S4).

### Different Correlation Patterns between the Various Plasma Lipid Classes and CVD Traditional Risk Factors

Partial correlations were performed between baseline lipid species levels and CVD risk factors (Table 4 and Supplementary material online, Figure S3) and the q-values for the statistically significant associations were good (1.29E-37≤q≤0.03) (Supplementary material online, Table S5). Few lipid species were correlated with carotid IMT, which in itself has previously been shown to predict incident coronary events, independently of cardiovascular risk factors [16], but all LPC species except one displayed negative correlation with the carotid IMT (*P≤*0.03). Correlation to the percentage of hemoglobin A1c (HbA1c) was seen mainly for the LPC and TAG species, with the former being negatively correlated (*P≤*0.01) and the later positively correlated (*P≤*0.04). Positive correlation to both LDL and HDL-cholesterol was observed for the majority of the glycerophospholipids with the exception of the LPC species which were only correlated to HDL-cholesterol (*P≤*0.04) (Supplementary material online, Figure S3).

**Table 4:** LPC16∶0 and LPC20∶4 negatively correlate with CVD risk factors whereas SM38∶2 positively correlates with CVD risk factors.

Lipid specie	LPC16∶0			LPC20∶4			SM38∶2		
	Correlation	*P*	q-value	Correlation	*P*	q-value	Correlation	*P*	q-value
Imtcca0	**−0.13**	**0.010**	**0.007**	**−**0.03	0.591	0.218	0.09	0.112	0.062
HbA1c	**−0.12**	**0.012**	**0.009**	**−**0.09	0.074	0.043	**−**0.05	0.385	0.163
BMI	**−0.14**	**0.004**	**0.003**	**−0.19**	**2.0E-04**	**2.2E-04**	0.04	0.438	0.180
SBP	**−**0.06	0.244	0.114	**−0.11**	**0.039**	**0.025**	**−**0.02	0.710	0.247
LDL	0.07	0.154	0.081	**−**0.05	0.356	0.153	**0.36**	**7.4E-11**	**2.6E-09**
HDL	**0.12**	**0.014**	**0.011**	**0.18**	**5.5E-04**	**5.8E-04**	0.06	0.262	0.121

Partial correlations were performed between LPC16∶0, LPC20∶4, or SM38∶2 after log transformation and current known laboratory predictors for cardiovascular disease, adjusting for age and sex. BMI, body mass index; HbA1c, haemoglobin A1c; HDL, high-density lipoprotein cholesterol; Imtcca0, intima-media thickness of the common carotid artery at baseline; LDL, low-density lipoprotein cholesterol; LPC, lysophosphatidylcholine; SBP, systolic blood pressure; SM, sphingomyelin.

The two CVD-protective LPC species, i.e., LPC16∶0 and LPC20∶4, were negatively correlated with BMI (*P≤*0.004) and positively correlated with HDL-cholesterol (*P≤*0.014) (Table 4). LPC16∶0, but not LPC20∶4, was negatively related to the carotid IMT and the percentage of HbA1c (*P≤*0.012). Also, LPC20∶4 was negatively associated with SBP (*P* = 0.039). SM38∶2, with a borderline *P*-value for increased association of future CVD odds, was positively correlated with LDL-cholesterol (*P* = 7.4*10^−11^).

### Association between Susceptibility Gene Variants for Coronary Artery Disease (CAD) and Plasma Lipid Profile

**Figure 2:**
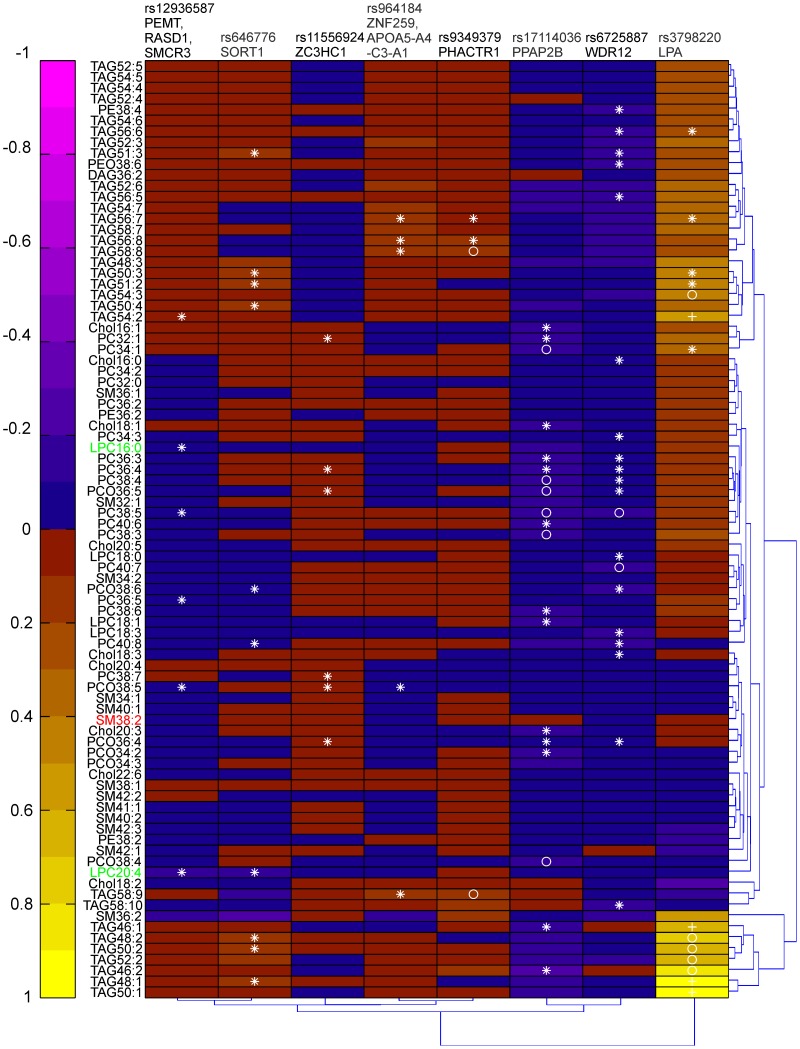
Association between the lipid profile and the risk allele of 8 CAD-associated gene variants. Heat map of regression coefficients obtained from linear regressions performed between the CAD-associated locus (with the CAD-associated allele coded) and the lipid species after log transformation adjusting for age and sex. *P<0.05, ^o^ *P*<0.01, ^+^ *P*<0.001.

We examined the association of 23 well-validated CAD-associated gene variants with circulating concentrations of the various lipid species, including the one associating with CVD outcome (Supplementary material online, Table S6). Eight of the gene variants displayed statistically significant association with several lipid species (Supplementary material online, Table S7) and the lipid pattern associated with those loci is depicted in Figure 2. However, the q-values were high for many of the associations (0.015≤q≤0.75) (Supplementary material online, Table S8). The CAD-associated risk allele for the LPA gene variant distinguished itself by being strongly associated with increased baseline plasma level of a cluster of TAG species composed by saturated/monounsaturated fatty acids. The risk allele for the WDR12, PPAP2B, SORT1 and PEMT/RASD1/SMCR3 loci were mainly associated with decreased baseline plasma level of glycerophospholipids, i.e. LPC, PC, PC-O, PE, PE-O, although SORT1 was also correlated with increased levels of several TAGs enriched in saturated/monounsaturated fatty acids. There was no clear association between any of the gene variants and the SM lipid species (Figure 2).

Carriers of the PEMT/RASD1/SMCR3 CAD risk allele had reduced level of the CVD-protective lipid specie LPC16∶0 (*P* = 0.031) as well as carriers of the PPAP2B CAD risk allele but the later association was only borderline significant (*P* = 0.056). Moreover, both carriers of the SORT1 and of the PEMT/RASD1/SMCR3 risk allele had reduced level of the CVD-protective lipid specie LPC20∶4 (*P* = 0.012 and *P* = 0.046, respectively). No association was found between any of the 23 CAD risk alleles and plasma level of SM38∶2 (Figure 2 and Supplementary material online, Table S7).

## Discussion

### Top-down Lipidomics, a Tool for Clinical Screens

The importance of two main lipids, i.e. triglycerides and cholesterol, as a tool for CVD prediction has long been known. But, modern lipidomics analysis shows that the human plasma lipidome comprises of at least several hundreds of individual lipid species and gives a glimpse of the complexity of the lipidome that has been overlooked until recently mainly because of technical limitations. We here performed a plasma lipidome screen in a prospective population-based cohort using top-down shotgun lipidomics. We aim to look for differences in the plasma composition in individuals with similar plasma total lipids level. We analyzed 427 samples with 2 technical replicates and identified and quantified 85 lipid species belonging to 9 different lipid classes and to our knowledge this study constitutes the first extensive lipid profiling of plasma for incident CVD in the primary preventive setting. Top-down shotgun lipidomics was the method of choice for this study because it is a quantitative and highly sensitive technique that allows high-throughput and relatively extensive lipid coverage.

### Refining the Dyslipidemia Phenotype

Although total increased plasma TAG concentration is considered a risk factor for CVD, we have here identified some individual TAG species that were associated with decreased odds of future CVD. LPC as a whole lipid class has previously been linked with inflammation as well as with both pro- and anti-atherogenic effects [26], [27], whereas we have here shown that two specific LPC species i.e., LPC16∶0 and LPC20∶4, were protective for CVD. These findings demonstrate that systematic analysis of plasma lipid species rather than lipid classes as a whole may reveal opposite relationships with CVD risk and thus could help to better understand the mechanisms leading to CVD and to improve CVD risk prediction.

Recently, a plasma lipidomics analysis was conducted using a different MS platform than the one we used, in a cross sectional setting, showing that certain patterns of lipids could discriminate between patients with stable angina and those with unstable CAD as well as healthy controls [9]. The results obtained in patients with unstable CAD are supported and extended by our prospective study of subjects without prior CVD, i.e., decreased level of most measured LPC species both in CAD versus control as well as in unstable versus stable CAD, increased levels of several SM species in unstable versus stable CAD and decreased level of specific TAG species in unstable versus stable CAD, were reported. This suggests that such alterations of lipid patterns may not only be a marker of coronary atherosclerosis and plaque instability but also that it may play a role in the pathogenesis of CVD, given its presence more than 10 years before clinical disease onset.

### Integrating Genomic and Lipidomics Information

Out of the 8 CAD susceptibility gene variants displaying significant association with circulating lipid species concentrations, 3 have not yet been previously reported to be involved in lipid metabolism (WDR12, ZC3HC1 and PHACTR1) and 3 are only known to affect lipoproteins levels (LPA, SORT1 and the ZNF259/APOA5-A4-C3-A1 gene region) [13], [28]. However, any potential link between the genetic alteration of these lipids and CAD needs to be substantiated by mechanistic studies. Two of the 8 CAD loci are directly coding for enzymes involved in lipids biosynthesis (PPAP2B and the PEMT/RASD1/SMCR3 locus) [29], [30]. The PPAP2B gene encodes a phosphatidic phosphatase that coverts phosphatidic acid into diacylglycerol, the precursor for *de novo* synthesis of TAG, PC and PE. Moreover, PEMT encodes an enzyme which sequentially converts PE into PC. Both carriers of the PPAP2B and of the PEMT/RASD1/SMCR3 risk allele display reduced level of multiple glycerophospholipids including the CVD-protective lipid species LPC16∶0 and/or LPC20∶4. Overall, our findings highlight that integrating lipidomics with genomics is a promising approach to increase the understanding of mechanisms underlying the gene-CVD associations as well as CVD pathogenesis.

### Study Limitations

This is an initial discovery study that needs to be replicated especially since the false discovery rate was high when looking for associations between the lipid species and future cardiovascular events or between the lipid species and most of the CAD-associated gene variants. Also, we do acknowledge that this is a case control study and not a general population study, thus the findings cannot be generalised to the whole population.Furthermore, our study could be complemented by acquiring spectra in negative ion mode to extend the lipid class coverage and by performing tandem MS for some targeted lipid species in order to get their full structural information. Another draw-back of the study is the lack of a pooled quality control plasma sample run across the study. Finally, we do not know to what extent the −80 degree Celsius storage over approximately 20 years may have affected the original lipid profile.

### Conclusions

This study constitutes a proof-of-concept screen that shotgun lipidomics can be used as a tool in the search for novel CVD biomarkers. Moreover, we here highlight the importance of refining the dyslipidemia phenotype and thus looking at the level of individual lipid species rather than the total sum of the different lipid classes in their relationship with CVD risk. We identified some specific lipid species as potential biomarkers of adverse cardiovascular outcome. However, statistical significance was lost for the association between the lipid species and future cardiovascular events when correcting for multiple testing. Finally, our results support the informative value in bringing together genomic and lipidomics data, suggesting that certain individual lipid species are intermediate phenotypes between genetic susceptibility and overt CVD. Overall, this is an explorative study that will need to be replicated in a larger population.

## Supporting Information

Figure S1 **Representative mass spectra of total lipid extracts from plasma.** The most abundant peaks are annotated with m/z; the shaded areas indicate the m/z ranges where the corresponding lipid classes were detected.
(PDF)

Figure S2 **Absolute quantification of TAGs by top-down lipidomics correlates with the total triglyceride levels measured at baseline examination.** Linear regression was performed between the total absolute TAG levels determined by MS versus the total triglyceride levels measured by traditional clinical chemistry analysis. The total TAG level measured by MS is obtained by summing the abundances of all the individual TAG species.
(PPT)

Figure S3 **Different correlation patterns between the various plasma lipid classes and CVD traditional risk factors.** Heat map of correlations coefficients obtained from partial correlations performed between the lipid species after log transformation and traditional laboratory predictors for cardiovascular disease adjusting for age and sex. *P<0.05, ^o^ *P*<0.01, ^+^ *P*<0.001.
(TIF)

Table S1 **Coefficient of variation (CV) of the combined lipid extraction and MS analysis for the 8 internal standards.**
(DOCX)

Table S2 **Absolute levels of the lipid species.**
(DOCX)

Table S3 A. Relation of baseline lipid specie level to future adverse cardiovascular outcome adjusting for Framingham risk factors. B. Relation of baseline lipid specie level to future adverse cardiovascular outcome adjusting for type 2diabetes only.
(DOCX)

Table S4 **Relation of baseline triglycerides specie level to future adverse cardiovascular outcome adjusting for Framingham risk factors.**
(DOCX)

Table S5 **Estimated q-values of the tests performed to study the association between CVD risk factors and the lipid species.**
(DOCX)

Table S6 **Relation between 23 validated coronary artery disease associated gene variants and baseline plasma lipid metabolites level.**
(DOCX)

Table S7 **The risk allele of 8 of the validated coronary artery disease associated gene variants shows significant association with the baseline plasma level of several lipid species.**
(DOCX)

Table S8 **Estimated q-values of the tests performed to study the association between CAD-associated gene variants and the lipid species.**
(DOCX)

## References

[1] Greenland P, Knoll MD, Stamler J, Neaton JD, Dyer AR, et al (2003) Major risk factors as antecedents of fatal and nonfatal coronary heart disease events. JAMA 290: 891–897. 12928465 10.1001/jama.290.7.891

[2] Khot UN, Khot MB, Bajzer CT, Sapp SK, Ohman EM, et al (2003) Prevalence of conventional risk factors in patients with coronary heart disease. JAMA 290: 898–904. 12928466 10.1001/jama.290.7.898

[3] Melander O, Newton-Cheh C, Almgren P, Hedblad B, Berglund G, et al (2009) Novel and conventional biomarkers for prediction of incident cardiovascular events in the community. JAMA 302: 49–57. 19567439 10.1001/jama.2009.943 PMC3090639

[4] Wang TJ, Gona P, Larson MG, Tofler GH, Levy D, et al (2006) Multiple biomarkers for the prediction of first major cardiovascular events and death. N Engl J Med 355: 2631–2639. 17182988 10.1056/NEJMoa055373

[5] Gross RW, Han X (2011) Lipidomics at the interface of structure and function in systems biology. Chem Biol 18: 284–291. 21439472 10.1016/j.chembiol.2011.01.014 PMC3132894

[6] Han X, Gross RW (2005) Shotgun lipidomics: electrospray ionization mass spectrometric analysis and quantitation of cellular lipidomes directly from crude extracts of biological samples. Mass Spectrom Rev 24: 367–412. 15389848 10.1002/mas.20023

[7] Sandra K, Pereira Ados S, Vanhoenacker G, David F, Sandra P (2010) Comprehensive blood plasma lipidomics by liquid chromatography/quadrupole time-of-flight mass spectrometry. J Chromatogr A 1217: 4087–4099. 20307888 10.1016/j.chroma.2010.02.039

[8] Graessler J, Schwudke D, Schwarz PE, Herzog R, Shevchenko A, et al (2009) Top-down lipidomics reveals ether lipid deficiency in blood plasma of hypertensive patients. PLoS One 4: e6261. 19603071 10.1371/journal.pone.0006261 PMC2705678

[9] Meikle PJ, Wong G, Tsorotes D, Barlow CK, Weir JM, et al (2011) Plasma lipidomic analysis of stable and unstable coronary artery disease. Arterioscler Thromb Vasc Biol 31: 2723–2732. 21903946 10.1161/ATVBAHA.111.234096

[10] Rhee EP, Cheng S, Larson MG, Walford GA, Lewis GD, et al (2011) Lipid profiling identifies a triacylglycerol signature of insulin resistance and improves diabetes prediction in humans. J Clin Invest 121: 1402–1411. 21403394 10.1172/JCI44442 PMC3069773

[11] Griffiths WJ, Ogundare M, Williams CM, Wang Y (2011) On the future of “omics”: lipidomics. J Inherit Metab Dis 34: 583–592. 21318352 10.1007/s10545-010-9274-4

[12] Fernandez C, Schuhmann K, Herzog R, Fielding B, Frayn K, et al (2011) Altered desaturation and elongation of fatty acids in hormone-sensitive lipase null mice. PLoS One 6: e21603. 21738729 10.1371/journal.pone.0021603 PMC3126817

[13] Schunkert H, Konig IR, Kathiresan S, Reilly MP, Assimes TL, et al (2011) Large-scale association analysis identifies 13 new susceptibility loci for coronary artery disease. Nat Genet 43: 333–338. 21378990 10.1038/ng.784 PMC3119261

[14] Berglund G, Elmstahl S, Janzon L, Larsson SA (1993) The Malmo Diet and Cancer Study. Design and feasibility. J Intern Med 233: 45–51. 8429286 10.1111/j.1365-2796.1993.tb00647.x

[15] Persson M, Hedblad B, Nelson JJ, Berglund G (2007) Elevated Lp-PLA2 levels add prognostic information to the metabolic syndrome on incidence of cardiovascular events among middle-aged nondiabetic subjects. Arterioscler Thromb Vasc Biol 27: 1411–1416. 17431184 10.1161/ATVBAHA.107.142679

[16] Rosvall M, Janzon L, Berglund G, Engstrom G, Hedblad B (2005) Incident coronary events and case fatality in relation to common carotid intima-media thickness. J Intern Med 257: 430–437. 15836659 10.1111/j.1365-2796.2005.01485.x

[17] Kathiresan S, Melander O, Anevski D, Guiducci C, Burtt NP, et al (2008) Polymorphisms associated with cholesterol and risk of cardiovascular events. N Engl J Med 358: 1240–1249. 18354102 10.1056/NEJMoa0706728

[18] Myocardial Infarction Genetics C, Kathiresan S, Voight BF, Purcell S, Musunuru K, et al (2009) Genome-wide association of early-onset myocardial infarction with single nucleotide polymorphisms and copy number variants. Nat Genet 41: 334–341. 19198609 10.1038/ng.327 PMC2681011

[19] Erdmann J, Grosshennig A, Braund PS, Konig IR, Hengstenberg C, et al (2009) New susceptibility locus for coronary artery disease on chromosome 3q22.3. Nat Genet 41: 280–282. 19198612 10.1038/ng.307 PMC2695543

[20] Ripatti S, Tikkanen E, Orho-Melander M, Havulinna AS, Silander K, et al (2010) A multilocus genetic risk score for coronary heart disease: case-control and prospective cohort analyses. Lancet 376: 1393–1400. 20971364 10.1016/S0140-6736(10)61267-6 PMC2965351

[21] Musunuru K, Orho-Melander M, Caulfield MP, Li S, Salameh WA, et al (2009) Ion mobility analysis of lipoprotein subfractions identifies three independent axes of cardiovascular risk. Arterioscler Thromb Vasc Biol 29: 1975–1980. 19729614 10.1161/ATVBAHA.109.190405 PMC2772123

[22] D’Agostino RB Sr, Grundy S, Sullivan LM, Wilson P (2001) Validation of the Framingham coronary heart disease prediction scores: results of a multiple ethnic groups investigation. JAMA 286: 180–187. 11448281 10.1001/jama.286.2.180

[23] Matyash V, Liebisch G, Kurzchalia TV, Shevchenko A, Schwudke D (2008) Lipid extraction by methyl-tert-butyl ether for high-throughput lipidomics. J Lipid Res 49: 1137–1146. 18281723 10.1194/jlr.D700041-JLR200 PMC2311442

[24] Herzog R, Schwudke D, Schuhmann K, Sampaio JL, Bornstein SR, et al (2011) A novel informatics concept for high-throughput shotgun lipidomics based on the molecular fragmentation query language. Genome Biol 12: R8. 21247462 10.1186/gb-2011-12-1-r8 PMC3091306

[25] Storey JD (2002) A direct approach to false discovery rates. Journal of the Royal Statistical Society Series B-Statistical Methodology 64: 479–498.

[26] Quinn MT, Parthasarathy S, Steinberg D (1988) Lysophosphatidylcholine: a chemotactic factor for human monocytes and its potential role in atherogenesis. Proc Natl Acad Sci U S A 85: 2805–2809. 3357891 10.1073/pnas.85.8.2805 PMC280088

[27] Schmitz G, Ruebsaamen K (2010) Metabolism and atherogenic disease association of lysophosphatidylcholine. Atherosclerosis 208: 10–18. 19570538 10.1016/j.atherosclerosis.2009.05.029

[28] Musunuru K, Strong A, Frank-Kamenetsky M, Lee NE, Ahfeldt T, et al (2010) From noncoding variant to phenotype via SORT1 at the 1p13 cholesterol locus. Nature 466: 714–719. 20686566 10.1038/nature09266 PMC3062476

[29] Kai M, Wada I, Imai S, Sakane F, Kanoh H (1997) Cloning and characterization of two human isozymes of Mg2+-independent phosphatidic acid phosphatase. J Biol Chem 272: 24572–24578. 9305923 10.1074/jbc.272.39.24572

[30] Walkey CJ, Donohue LR, Bronson R, Agellon LB, Vance DE (1997) Disruption of the murine gene encoding phosphatidylethanolamine N-methyltransferase. Proc Natl Acad Sci U S A 94: 12880–12885. 9371769 10.1073/pnas.94.24.12880 PMC24232

