# Supplementary material for: Plasma Lipid Composition and Risk of Developing Cardiovascular Disease
Source: PLoS One. 2013 Aug 15;8(8):e71846. doi: 10.1371/journal.pone.0071846 (PMC3744469; doi:10.1371/journal.pone.0071846)

Supplementary Figure S1A

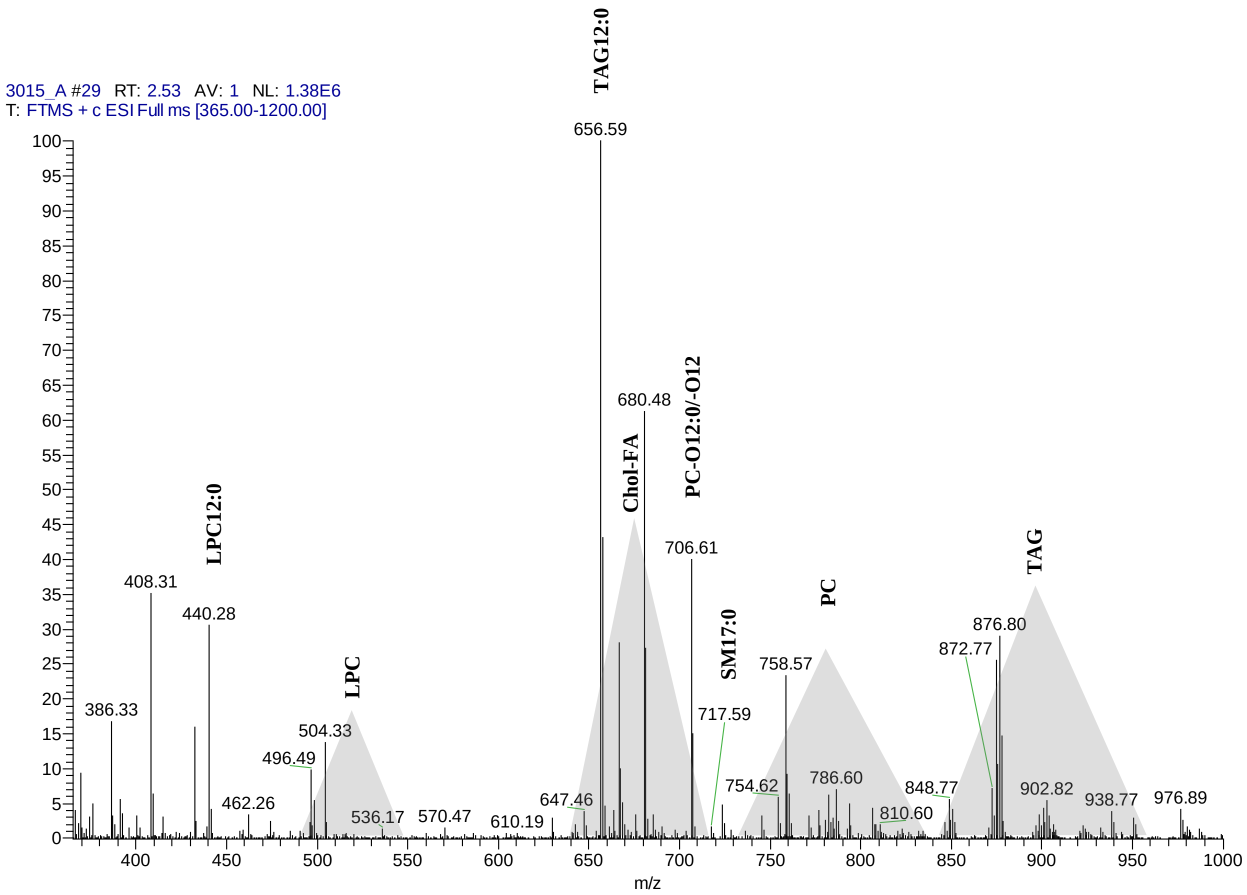

Supplementary Figure S1B

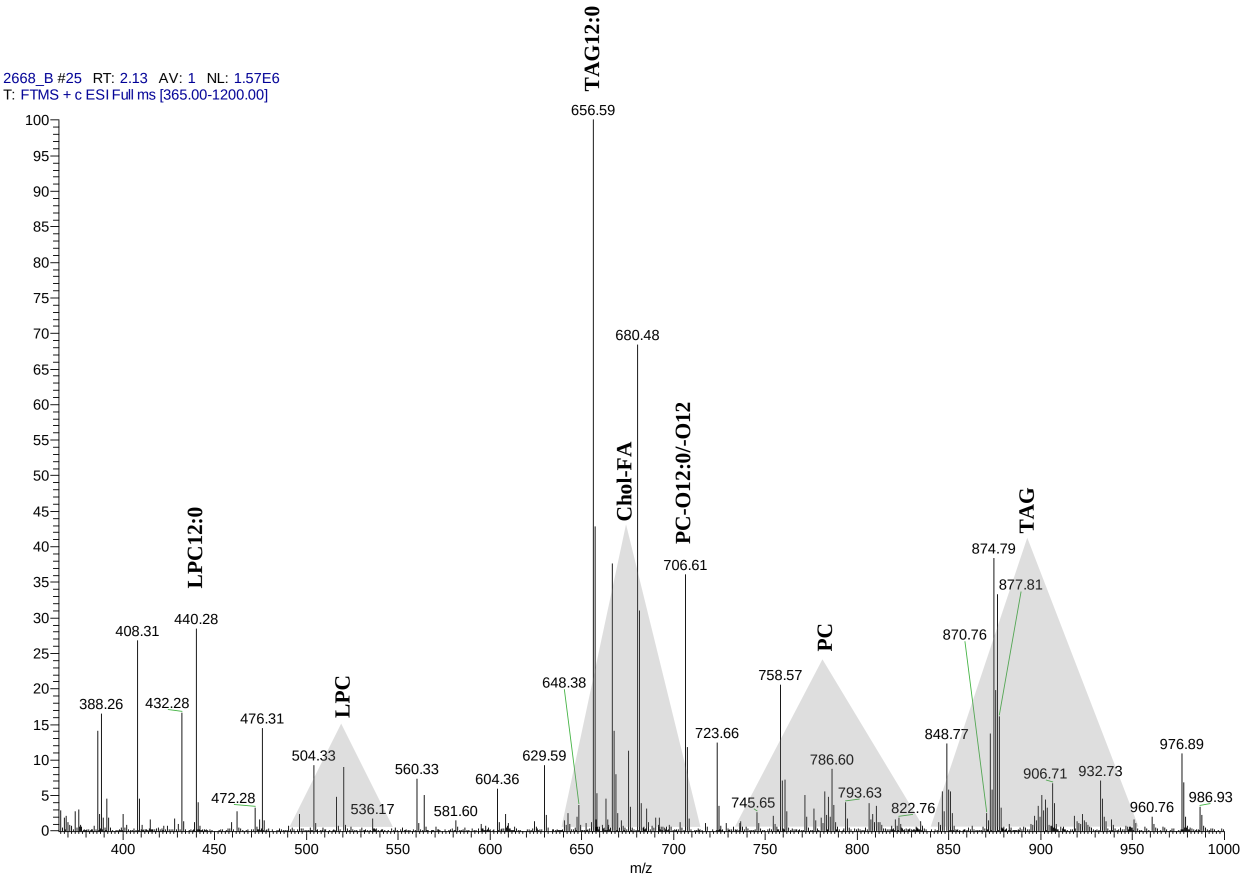

Supplement: Figure S1 — Representative mass spectra of total lipid extracts from plasma. The most abundant peaks are annotated with m/z; the shaded areas indicate the m/z ranges where the corresponding lipid classes were detected. (PDF) [file pone.0071846.s001.pdf]
