# Supplementary figures and images for: Plasma Lipid Composition and Risk of Developing Cardiovascular Disease
Source: PLoS One. 2013 Aug 15;8(8):e71846. doi: 10.1371/journal.pone.0071846 (PMC3744469; doi:10.1371/journal.pone.0071846)

## Slide 1
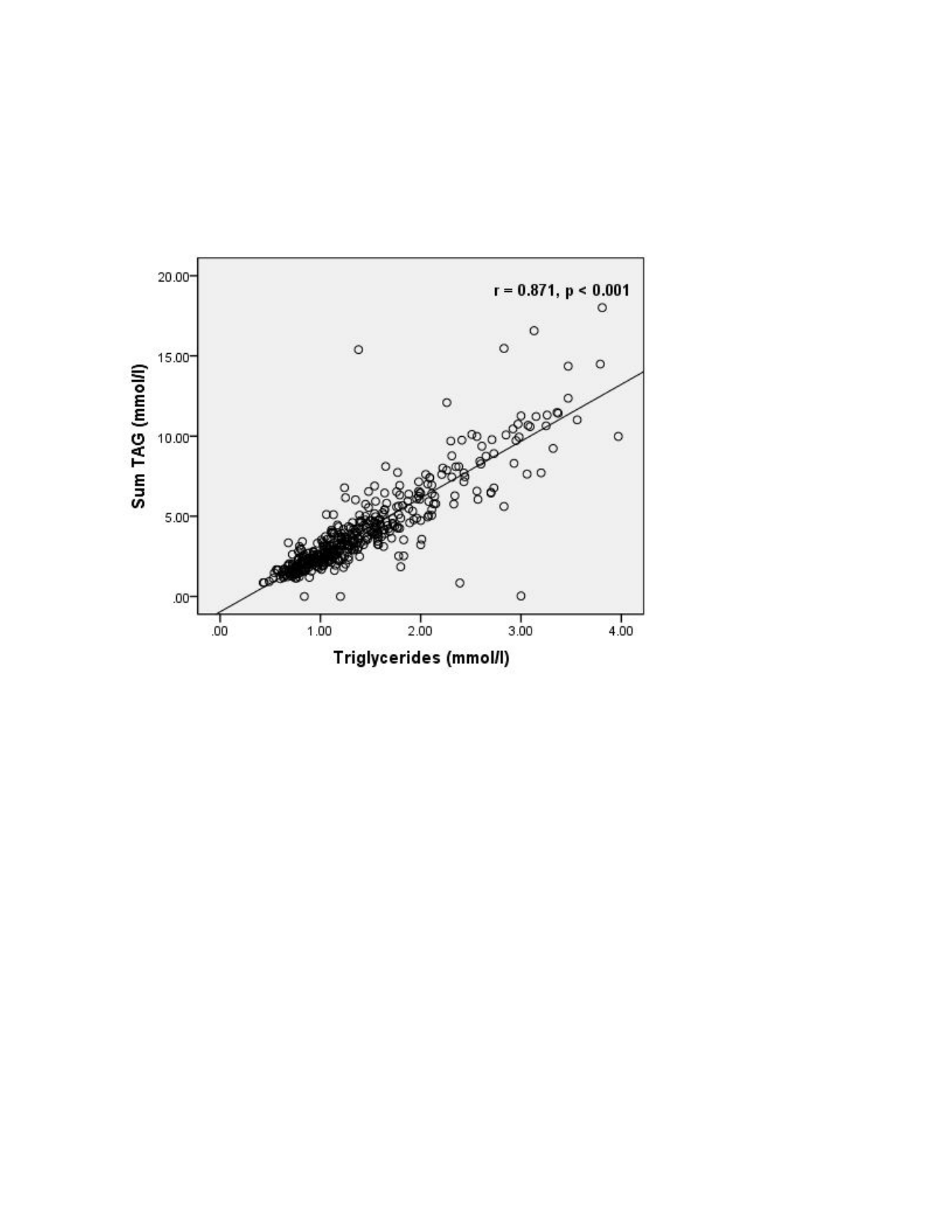

Supplement: Figure S2 — Absolute quantification of TAGs by top-down lipidomics correlates with the total triglyceride levels measured at baseline examination. Linear regression was performed between the total absolute TAG levels determined by MS versus the total triglyceride levels measured by traditional clinical chemistry analysis. The total TAG level measured by MS is obtained by summing the abundances of all the individual TAG species. (PPT) [file pone.0071846.s002.ppt]

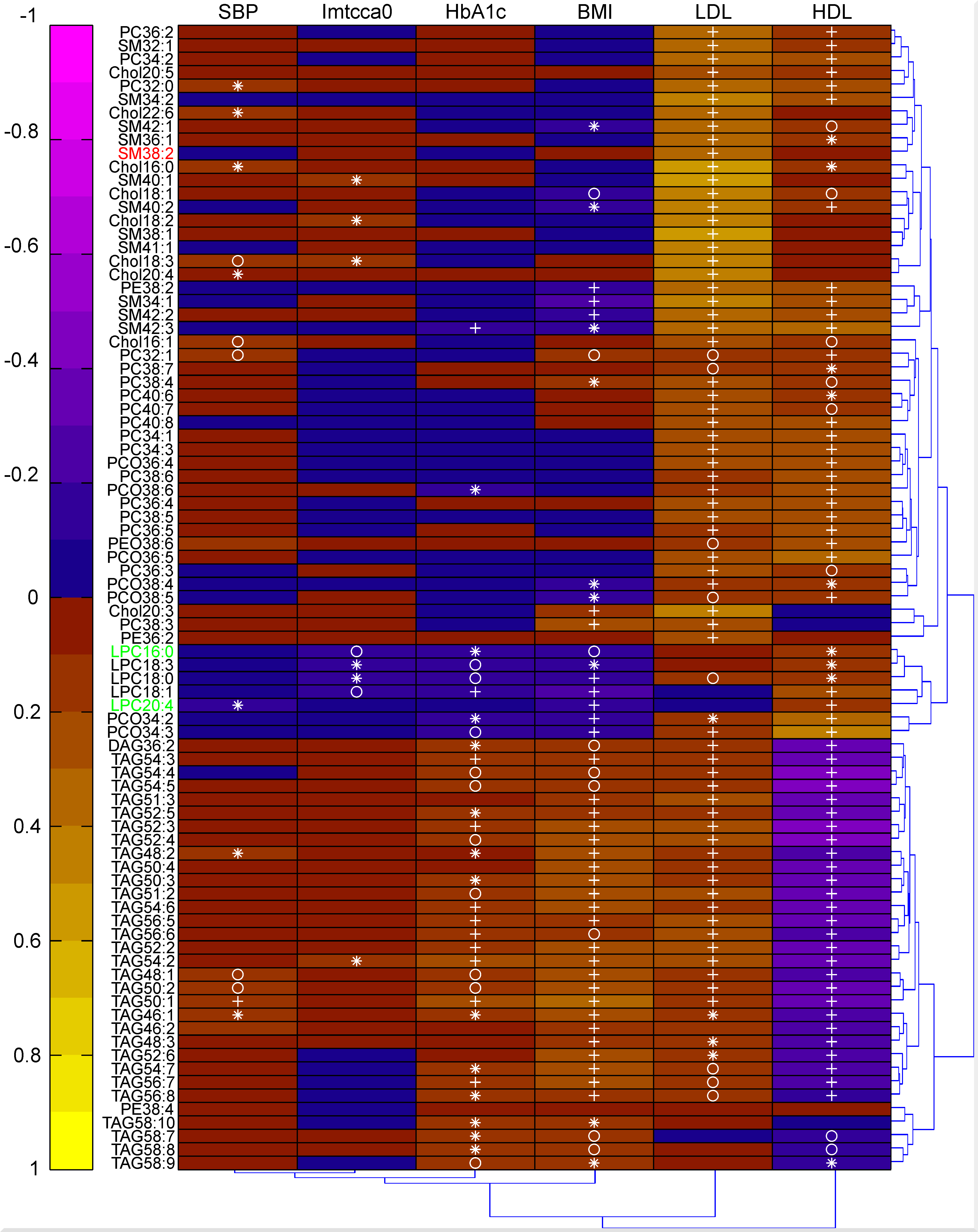

Supplement: Figure S3 — Different correlation patterns between the various plasma lipid classes and CVD traditional risk factors. Heat map of correlations coefficients obtained from partial correlations performed between the lipid species after log transformation and traditional laboratory predictors for cardiovascular disease adjusting for age and sex. *P<0.05, o P<0.01, + P<0.001. (TIF) [file pone.0071846.s003.tif]
