# Supplementary material for: Plasma Lipid Composition and Risk of Developing Cardiovascular Disease
Source: PLoS One. 2013 Aug 15;8(8):e71846. doi: 10.1371/journal.pone.0071846 (PMC3744469; doi:10.1371/journal.pone.0071846)
Supplement: Table S1 — Coefficient of variation (CV) of the combined lipid extraction and MS analysis for the 8 internal standards. (DOCX) [file pone.0071846.s004.docx]

**Supplementary Table S1.** Coefficient of variation (CV) of the combined lipid extraction and MS analysis for the 8 internal standards

|  | CV |
| --- | --- |
| CE17:0 | 34% |
| SM17:0 | 30% |
| PC-O12:0/-O12:0 | 26% |
| PE-O16:0/-O16:0 | 35% |
| LPC12:0 | 25% |
| Cer17:0 | 26% |
| TAG12:0 | 20% |
| DAG12:0 | 32% |

CE, cholesterylester; Cer, ceramide; DAG, diacylglyceride; LPC, lysophosphatidylcholine; PC-O, phosphatidylcholine ether; PE-O, phosphatidylethanolamine ether; SM, sphingomyelin; TAG, triacylglyceride.
