# Supplementary material for: Plasma Lipid Composition and Risk of Developing Cardiovascular Disease
Source: PLoS One. 2013 Aug 15;8(8):e71846. doi: 10.1371/journal.pone.0071846 (PMC3744469; doi:10.1371/journal.pone.0071846)
Supplement: Table S2 — Absolute levels of the lipid species. (DOCX) [file pone.0071846.s005.docx]

**Supplementary Table S2.** Absolute levels of the lipid species

|  | Control | | CVD case | |
| --- | --- | --- | --- | --- |
|  | Mean (pmol/mL) | SEM | Mean (pmol/mL) | SEM |
| Chol 16:0 | 4179.0 | 89.1 | 4164.4 | 90.9 |
| Chol 16:1 | 1722.7 | 62.8 | 1745.4 | 68.9 |
| Chol 18:1 | 11207.9 | 295.6 | 11561.2 | 334.4 |
| Chol 18:2 | 65793.7 | 1540.7 | 66514.1 | 1535.7 |
| Chol 18:3 | 920.9 | 23.9 | 940.5 | 25.6 |
| Chol 20:3 | 307.0 | 9.7 | 304.9 | 9.6 |
| Chol 20:4 | 3627.5 | 92.1 | 3647.6 | 98.5 |
| Chol 20:5 | 1064.7 | 52.5 | 1017.8 | 44.8 |
| Chol 22:6 | 529.8 | 17.1 | 514.3 | 15.9 |
| DAG 36:2 | 69.9 | 3.5 | 64.2 | 3.6 |
| LPC 16:0 | 573.0 | 20.9 | 526.9 | 22.0 |
| LPC 18:0 | 156.7 | 4.9 | 144.7 | 4.8 |
| LPC 18:1 | 119.8 | 4.1 | 109.8 | 3.6 |
| LPC 18:3 | 59.7 | 2.4 | 55.7 | 2.3 |
| LPC 20:4 | 22.9 | 1.6 | 17.9 | 1.2 |
| PC 32:0 | 59.3 | 1.2 | 58.3 | 1.2 |
| PC 32:1 | 89.7 | 3.9 | 89.2 | 4.1 |
| PC 34:1 | 1634.1 | 53.9 | 1667.4 | 52.7 |
| PC 34:2 | 4966.0 | 123.0 | 4966.5 | 111.8 |
| PC 34:3 | 99.8 | 2.5 | 99.0 | 2.6 |
| PC 36:2 | 1859.8 | 48.4 | 1842.2 | 46.9 |
| PC 36:3 | 915.6 | 23.0 | 906.5 | 22.9 |
| PC 36:4 | 1303.8 | 36.3 | 1290.0 | 33.7 |
| PC 36:5 | 421.2 | 19.1 | 404.3 | 16.2 |
| PC 38:3 | 187.6 | 5.4 | 182.1 | 5.1 |
| PC 38:4 | 554.7 | 13.8 | 549.2 | 12.9 |
| PC 38:5 | 436.7 | 12.9 | 419.2 | 10.9 |
| PC 38:6 | 870.6 | 29.7 | 815.3 | 23.1 |
| PC 38:7 | 93.7 | 3.8 | 89.3 | 3.6 |
| PC 40:6 | 231.5 | 6.8 | 218.1 | 5.7 |
| PC 40:7 | 74.4 | 2.1 | 70.9 | 1.9 |
| PC 40:8 | 31.4 | 1.2 | 29.2 | 0.9 |
| PC-O 34:2 | 25.9 | 0.9 | 27.5 | 1.1 |
| PC-O 34:3 | 23.7 | 0.9 | 24.9 | 1.0 |
| PC-O 36:4 | 64.2 | 1.5 | 63.9 | 1.5 |
| PC-O 36:5 | 47.0 | 1.3 | 46.8 | 1.2 |
| PC-O 38:4 | 28.2 | 0.8 | 27.7 | 0.9 |
| PC-O 38:5 | 72.5 | 1.8 | 74.9 | 1.9 |
| PC-O 38:6 | 41.5 | 1.4 | 41.3 | 1.6 |
| PE 36:2 | 45.3 | 1.9 | 43.4 | 1.7 |
| PE 38:2 | 129.5 | 3.5 | 125.6 | 3.3 |
| PE 38:4 | 38.6 | 2.0 | 36.4 | 1.7 |
| PE-O 38:6 | 27.8 | 1.7 | 28.1 | 1.6 |
| SM 32:1 | 135.0 | 4.5 | 141.8 | 4.8 |
| SM 34:1 | 1745.6 | 30.5 | 1788.6 | 32.8 |
| SM 34:2 | 250.8 | 5.8 | 252.2 | 5.8 |
| SM 36:1 | 310.4 | 8.8 | 315.9 | 8.6 |
| SM 38:1 | 237.0 | 5.3 | 242.7 | 5.4 |
| SM 38:2 | 56.4 | 3.5 | 61.2 | 3.5 |
| SM 40:1 | 456.7 | 9.4 | 456.5 | 9.0 |
| SM 40:2 | 426.8 | 9.2 | 424.7 | 8.8 |
| SM 41:1 | 151.5 | 4.5 | 152.7 | 4.8 |
| SM 42:1 | 178.6 | 6.6 | 180.8 | 6.6 |
| SM 42:2 | 912.6 | 15.8 | 928.0 | 16.7 |
| SM 42:3 | 427.3 | 8.3 | 425.8 | 8.4 |
| TAG 46:1 | 51.5 | 2.8 | 45.9 | 1.7 |
| TAG 46:2 | 12.5 | 1.3 | 9.8 | 0.8 |
| TAG 48:1 | 98.2 | 8.0 | 81.1 | 5.1 |
| TAG 48:2 | 75.0 | 4.7 | 63.9 | 3.5 |
| TAG 48:3 | 23.9 | 1.4 | 20.4 | 1.1 |
| TAG 50:1 | 216.2 | 17.7 | 184.7 | 12.8 |
| TAG 50:2 | 386.8 | 23.8 | 346.2 | 20.1 |
| TAG 50:3 | 158.7 | 7.2 | 141.6 | 6.0 |
| TAG 50:4 | 40.4 | 1.8 | 36.0 | 1.5 |
| TAG 51:2 | 25.1 | 1.0 | 22.2 | 0.9 |
| TAG 51:3 | 16.6 | 0.7 | 14.7 | 0.6 |
| TAG 52:2 | 916.3 | 50.7 | 857.5 | 48.8 |
| TAG 52:3 | 1051.5 | 53.8 | 965.7 | 47.5 |
| TAG 52:4 | 330.0 | 16.3 | 298.6 | 13.2 |
| TAG 52:5 | 69.8 | 2.8 | 64.0 | 2.4 |
| TAG 52:6 | 15.5 | 0.8 | 13.7 | 0.8 |
| TAG 54:2 | 41.7 | 1.7 | 40.1 | 1.5 |
| TAG 54:3 | 169.4 | 7.6 | 163.5 | 7.2 |
| TAG 54:4 | 167.1 | 7.2 | 159.8 | 6.7 |
| TAG 54:5 | 112.9 | 4.7 | 106.0 | 4.0 |
| TAG 54:6 | 65.9 | 2.7 | 61.1 | 2.4 |
| TAG 54:7 | 28.4 | 1.4 | 26.8 | 1.3 |
| TAG 56:5 | 21.0 | 0.8 | 20.6 | 0.7 |
| TAG 56:6 | 36.5 | 1.3 | 34.3 | 1.1 |
| TAG 56:7 | 70.1 | 3.3 | 67.6 | 3.1 |
| TAG 56:8 | 39.9 | 2.4 | 37.6 | 1.8 |
| TAG 58:7 | 5.9 | 0.4 | 5.5 | 0.5 |
| TAG 58:8 | 13.6 | 0.8 | 13.4 | 0.6 |
| TAG 58:9 | 8.6 | 0.7 | 8.8 | 0.5 |
| TAG 58:10 | 5.3 | 0.5 | 5.0 | 0.4 |

Chol, cholesterylester; DAG, diacylglyceride; LPC, lysophosphatidylcholine; PC, phosphatidyl-choline; PC-O, phosphatidylcholine ether; PE, phosphatidylethanolamine; PE-O, phosphatidylethanolamine ether; SM, sphingomyelin; TAG, triacylglyceride.
