# Supplementary material for: Plasma Lipid Composition and Risk of Developing Cardiovascular Disease
Source: PLoS One. 2013 Aug 15;8(8):e71846. doi: 10.1371/journal.pone.0071846 (PMC3744469; doi:10.1371/journal.pone.0071846)
Supplement: Table S3 — A. Relation of baseline lipid specie level to future adverse cardiovascular outcome adjusting for Framingham risk factors. B. Relation of baseline lipid specie level to future adverse cardiovascular outcome adjusting for type 2diabetes only. (DOCX) [file pone.0071846.s006.docx]

**Supplementary Table S3A.** Relation of baseline lipid specie level to future adverse cardiovascular outcome adjusting for Framingham risk factors

| Lipid specie | *P* | q-value | Odds ratio per s.d. | 95% CI (lower) | 95% CI (upper) |
| --- | --- | --- | --- | --- | --- |
| Chol 16:0 | 0.347 | 0.296 | 0.89 | 0.69 | 1.14 |
| Chol 16:1 | 0.532 | 0.300 | 0.93 | 0.75 | 1.16 |
| Chol 18:1 | 0.607 | 0.323 | 0.94 | 0.75 | 1.18 |
| Chol 18:2 | 0.991 | 0.408 | 1.00 | 0.80 | 1.26 |
| Chol 18:3 | 0.658 | 0.339 | 1.05 | 0.84 | 1.32 |
| Chol 20:3 | 0.751 | 0.346 | 0.96 | 0.77 | 1.20 |
| Chol 20:4 | 0.531 | 0.300 | 0.93 | 0.74 | 1.16 |
| Chol 20:5 | 0.578 | 0.321 | 0.94 | 0.76 | 1.16 |
| Chol 22:6 | 0.487 | 0.297 | 0.93 | 0.75 | 1.15 |
| DAG 36:2 | 0.123 | 0.237 | 0.84 | 0.67 | 1.05 |
| LPC 16:0 | 0.028 | 0.210 | 0.79 | 0.65 | 0.97 |
| LPC 18:0 | 0.094 | 0.237 | 0.84 | 0.68 | 1.03 |
| LPC 18:1 | 0.160 | 0.237 | 0.86 | 0.70 | 1.06 |
| LPC 18:3 | 0.227 | 0.264 | 0.88 | 0.72 | 1.08 |
| LPC 20:4 | 0.024 | 0.210 | 0.77 | 0.61 | 0.96 |
| PC 32:0 | 0.436 | 0.297 | 0.92 | 0.74 | 1.14 |
| PC 32:1 | 0.397 | 0.297 | 0.91 | 0.74 | 1.13 |
| PC 34:1 | 0.465 | 0.297 | 0.92 | 0.75 | 1.14 |
| PC 34:2 | 0.768 | 0.349 | 0.97 | 0.78 | 1.20 |
| PC 34:3 | 0.370 | 0.296 | 0.91 | 0.74 | 1.12 |
| PC 36:2 | 0.610 | 0.323 | 0.95 | 0.76 | 1.17 |
| PC 36:3 | 0.241 | 0.264 | 0.88 | 0.71 | 1.09 |
| PC 36:4 | 0.331 | 0.296 | 0.90 | 0.73 | 1.11 |
| PC 36:5 | 0.322 | 0.296 | 0.90 | 0.73 | 1.11 |
| PC 38:3 | 0.269 | 0.277 | 0.89 | 0.72 | 1.10 |
| PC 38:4 | 0.421 | 0.297 | 0.92 | 0.74 | 1.13 |
| PC 38:5 | 0.164 | 0.237 | 0.86 | 0.69 | 1.06 |
| PC 38:6 | 0.139 | 0.237 | 0.85 | 0.69 | 1.05 |
| PC 38:7 | 0.828 | 0.362 | 0.98 | 0.80 | 1.20 |
| PC 40:6 | 0.132 | 0.237 | 0.85 | 0.69 | 1.05 |
| PC 40:7 | 0.340 | 0.296 | 0.90 | 0.73 | 1.11 |
| PC 40:8 | 0.302 | 0.296 | 0.89 | 0.72 | 1.10 |
| PC-O 34:2 | 0.184 | 0.241 | 1.16 | 0.93 | 1.44 |
| PC-O 34:3 | 0.219 | 0.264 | 1.16 | 0.92 | 1.47 |
| PC-O 36:4 | 0.860 | 0.372 | 0.98 | 0.80 | 1.21 |
| PC-O 36:5 | 0.974 | 0.406 | 1.00 | 0.80 | 1.24 |
| PC-O 38:4 | 0.669 | 0.339 | 0.96 | 0.78 | 1.17 |
| PC-O 38:5 | 0.402 | 0.297 | 1.09 | 0.89 | 1.34 |
| PC-O 38:6 | 0.793 | 0.356 | 0.97 | 0.79 | 1.19 |
| PE 36:2 | 0.483 | 0.297 | 0.93 | 0.75 | 1.15 |
| PE 38:2 | 0.426 | 0.297 | 0.91 | 0.73 | 1.14 |
| PE 38:4 | 0.889 | 0.379 | 0.98 | 0.79 | 1.22 |
| PE-O 38:6 | 0.507 | 0.297 | 0.92 | 0.72 | 1.17 |
| SM 32:1 | 0.269 | 0.277 | 1.13 | 0.91 | 1.41 |
| SM 34:1 | 0.417 | 0.297 | 1.11 | 0.86 | 1.42 |
| SM 34:2 | 0.610 | 0.323 | 1.06 | 0.84 | 1.35 |
| SM 36:1 | 0.508 | 0.297 | 1.08 | 0.87 | 1.34 |
| SM 38:1 | 0.438 | 0.297 | 1.10 | 0.87 | 1.38 |
| SM 38:2 | 0.057 | 0.228 | 1.28 | 0.99 | 1.64 |
| SM 40:1 | 0.457 | 0.297 | 0.91 | 0.71 | 1.16 |
| SM 40:2 | 0.690 | 0.340 | 0.95 | 0.75 | 1.21 |
| SM 41:1 | 0.749 | 0.346 | 1.04 | 0.83 | 1.30 |
| SM 42:1 | 0.240 | 0.264 | 0.87 | 0.70 | 1.09 |
| SM 42:2 | 0.741 | 0.346 | 1.04 | 0.83 | 1.30 |
| SM 42:3 | 0.816 | 0.362 | 1.03 | 0.81 | 1.30 |
| TAG 46:1 | 0.100 | 0.237 | 0.84 | 0.68 | 1.03 |
| TAG 46:2 | 0.148 | 0.237 | 0.84 | 0.66 | 1.06 |
| TAG 48:1 | 0.031 | 0.210 | 0.78 | 0.63 | 0.98 |
| TAG 48:2 | 0.034 | 0.210 | 0.79 | 0.64 | 0.98 |
| TAG 48:3 | 0.049 | 0.228 | 0.81 | 0.65 | 1.00 |
| TAG 50:1 | 0.065 | 0.228 | 0.81 | 0.64 | 1.01 |
| TAG 50:2 | 0.074 | 0.235 | 0.82 | 0.65 | 1.02 |
| TAG 50:3 | 0.036 | 0.210 | 0.79 | 0.63 | 0.98 |
| TAG 50:4 | 0.033 | 0.210 | 0.79 | 0.64 | 0.98 |
| TAG 51:2 | 0.059 | 0.228 | 0.81 | 0.65 | 1.01 |
| TAG 51:3 | 0.356 | 0.296 | 0.90 | 0.72 | 1.12 |
| TAG 52:2 | 0.236 | 0.264 | 0.87 | 0.70 | 1.09 |
| TAG 52:3 | 0.186 | 0.241 | 0.86 | 0.68 | 1.08 |
| TAG 52:4 | 0.159 | 0.237 | 0.85 | 0.68 | 1.06 |
| TAG 52:5 | 0.145 | 0.237 | 0.85 | 0.68 | 1.06 |
| TAG 52:6 | 0.137 | 0.237 | 0.85 | 0.69 | 1.05 |
| TAG 54:2 | 0.462 | 0.297 | 0.92 | 0.74 | 1.15 |
| TAG 54:3 | 0.509 | 0.297 | 0.93 | 0.75 | 1.16 |
| TAG 54:4 | 0.469 | 0.297 | 0.92 | 0.74 | 1.15 |
| TAG 54:5 | 0.372 | 0.296 | 0.91 | 0.73 | 1.13 |
| TAG 54:6 | 0.161 | 0.237 | 0.86 | 0.69 | 1.06 |
| TAG 54:7 | 0.709 | 0.345 | 0.96 | 0.78 | 1.18 |
| TAG 56:5 | 0.905 | 0.382 | 1.01 | 0.82 | 1.25 |
| TAG 56:6 | 0.169 | 0.237 | 0.86 | 0.69 | 1.07 |
| TAG 56:7 | 0.648 | 0.339 | 0.95 | 0.77 | 1.17 |
| TAG 56:8 | 0.743 | 0.346 | 0.97 | 0.79 | 1.19 |
| TAG 58:7 | 0.130 | 0.237 | 0.83 | 0.65 | 1.06 |
| TAG 58:8 | 0.682 | 0.340 | 1.04 | 0.85 | 1.28 |
| TAG 58:9 | 0.359 | 0.296 | 1.11 | 0.89 | 1.37 |
| TAG 58:10 | 0.331 | 0.296 | 0.89 | 0.70 | 1.13 |

Values are odds ratios (95% confidence intervals) for cardiovascular disease from multivariate adjusted binary logistic regressions performed with the Z score of a given lipid specie obtained after log transformation. Chol, cholesterylester; DAG, diacylglyceride; LPC, lysophosphatidylcholine; PC, phosphatidyl-choline; PC-O, phosphatidylcholine ether; PE, phosphatidylethanolamine; PE-O, phosphatidylethanolamine ether; SM, sphingomyelin; TAG, triacylglyceride.

**Supplementary Table S3B.** Relation of baseline lipid specie level to future adverse cardiovascular outcome adjusting for type 2diabetes only

| Lipid specie | *P* | q-value | Odds ratio per s.d. | 95% CI (lower) | 95% CI (upper) |
| --- | --- | --- | --- | --- | --- |
| Chol 16:0 | 0.927 | 0.818 | 0.991 | 0.818 | 1.201 |
| Chol 16:1 | 0.938 | 0.818 | 0.992 | 0.818 | 1.204 |
| Chol 18:1 | 0.983 | 0.818 | 1.002 | 0.828 | 1.213 |
| Chol 18:2 | 0.651 | 0.781 | 1.045 | 0.862 | 1.267 |
| Chol 18:3 | 0.389 | 0.664 | 1.089 | 0.897 | 1.322 |
| Chol 20:3 | 0.941 | 0.818 | 1.007 | 0.831 | 1.221 |
| Chol 20:4 | 0.977 | 0.818 | 1.003 | 0.828 | 1.215 |
| Chol 20:5 | 0.948 | 0.818 | 0.994 | 0.820 | 1.204 |
| Chol 22:6 | 0.794 | 0.818 | 0.975 | 0.804 | 1.181 |
| DAG 36:2 | 0.076 | 0.448 | 0.829 | 0.673 | 1.020 |
| LPC 16:0 | 0.053 | 0.448 | 0.823 | 0.676 | 1.002 |
| LPC 18:0 | 0.160 | 0.487 | 0.869 | 0.715 | 1.057 |
| LPC 18:1 | 0.272 | 0.581 | 0.896 | 0.737 | 1.090 |
| LPC 18:3 | 0.292 | 0.581 | 0.900 | 0.739 | 1.095 |
| LPC 20:4 | 0.059 | 0.448 | 0.813 | 0.655 | 1.008 |
| PC 32:0 | 0.903 | 0.818 | 0.988 | 0.815 | 1.198 |
| PC 32:1 | 0.685 | 0.795 | 0.961 | 0.791 | 1.166 |
| PC 34:1 | 0.757 | 0.818 | 0.970 | 0.800 | 1.176 |
| PC 34:2 | 0.825 | 0.818 | 1.022 | 0.843 | 1.238 |
| PC 34:3 | 0.625 | 0.781 | 0.953 | 0.787 | 1.155 |
| PC 36:2 | 0.981 | 0.818 | 0.998 | 0.824 | 1.208 |
| PC 36:3 | 0.401 | 0.664 | 0.920 | 0.757 | 1.118 |
| PC 36:4 | 0.758 | 0.818 | 0.970 | 0.799 | 1.177 |
| PC 36:5 | 0.645 | 0.781 | 0.956 | 0.788 | 1.159 |
| PC 38:3 | 0.432 | 0.665 | 0.925 | 0.762 | 1.123 |
| PC 38:4 | 0.767 | 0.818 | 0.971 | 0.801 | 1.178 |
| PC 38:5 | 0.451 | 0.665 | 0.928 | 0.765 | 1.126 |
| PC 38:6 | 0.303 | 0.581 | 0.904 | 0.745 | 1.096 |
| PC 38:7 | 0.899 | 0.818 | 1.013 | 0.833 | 1.231 |
| PC 40:6 | 0.231 | 0.581 | 0.889 | 0.732 | 1.078 |
| PC 40:7 | 0.524 | 0.717 | 0.939 | 0.775 | 1.139 |
| PC 40:8 | 0.578 | 0.763 | 0.946 | 0.779 | 1.149 |
| PC-O 34:2 | 0.096 | 0.462 | 1.185 | 0.971 | 1.446 |
| PC-O 34:3 | 0.095 | 0.462 | 1.187 | 0.971 | 1.452 |
| PC-O 36:4 | 0.662 | 0.781 | 1.044 | 0.861 | 1.266 |
| PC-O 36:5 | 0.474 | 0.685 | 1.073 | 0.885 | 1.301 |
| PC-O 38:4 | 0.915 | 0.818 | 0.990 | 0.816 | 1.201 |
| PC-O 38:5 | 0.259 | 0.581 | 1.119 | 0.921 | 1.359 |
| PC-O 38:6 | 0.857 | 0.818 | 1.018 | 0.840 | 1.233 |
| PE 36:2 | 0.590 | 0.763 | 0.946 | 0.773 | 1.158 |
| PE 38:2 | 0.803 | 0.818 | 0.976 | 0.803 | 1.185 |
| PE 38:4 | 0.924 | 0.818 | 0.990 | 0.802 | 1.221 |
| PE-O 38:6 | 0.973 | 0.818 | 0.996 | 0.797 | 1.245 |
| SM 32:1 | 0.167 | 0.487 | 1.147 | 0.944 | 1.393 |
| SM 34:1 | 0.236 | 0.581 | 1.126 | 0.925 | 1.370 |
| SM 34:2 | 0.405 | 0.664 | 1.085 | 0.895 | 1.316 |
| SM 36:1 | 0.312 | 0.581 | 1.105 | 0.911 | 1.340 |
| SM 38:1 | 0.252 | 0.581 | 1.120 | 0.923 | 1.358 |
| SM 38:2 | 0.051 | 0.448 | 1.252 | 0.999 | 1.567 |
| SM 40:1 | 0.936 | 0.818 | 0.992 | 0.819 | 1.202 |
| SM 40:2 | 0.888 | 0.818 | 1.014 | 0.837 | 1.229 |
| SM 41:1 | 0.413 | 0.664 | 1.084 | 0.894 | 1.315 |
| SM 42:1 | 0.446 | 0.665 | 0.925 | 0.756 | 1.131 |
| SM 42:2 | 0.426 | 0.665 | 1.082 | 0.891 | 1.313 |
| SM 42:3 | 0.487 | 0.689 | 1.071 | 0.882 | 1.301 |
| TAG 46:1 | 0.111 | 0.462 | 0.850 | 0.695 | 1.038 |
| TAG 46:2 | 0.161 | 0.487 | 0.849 | 0.675 | 1.068 |
| TAG 48:1 | 0.036 | 0.448 | 0.804 | 0.656 | .986 |
| TAG 48:2 | 0.033 | 0.448 | 0.804 | 0.657 | .982 |
| TAG 48:3 | 0.043 | 0.448 | 0.811 | 0.662 | .993 |
| TAG 50:1 | 0.072 | 0.448 | 0.827 | 0.672 | 1.017 |
| TAG 50:2 | 0.067 | 0.448 | 0.827 | 0.675 | 1.013 |
| TAG 50:3 | 0.031 | 0.448 | 0.802 | 0.657 | .980 |
| TAG 50:4 | 0.024 | 0.448 | 0.795 | 0.652 | .970 |
| TAG 51:2 | 0.065 | 0.448 | 0.829 | 0.680 | 1.012 |
| TAG 51:3 | 0.308 | 0.581 | 0.903 | 0.741 | 1.099 |
| TAG 52:2 | 0.182 | 0.495 | 0.873 | 0.714 | 1.066 |
| TAG 52:3 | 0.133 | 0.471 | 0.859 | 0.705 | 1.047 |
| TAG 52:4 | 0.110 | 0.462 | 0.852 | 0.700 | 1.037 |
| TAG 52:5 | 0.104 | 0.462 | 0.850 | 0.698 | 1.034 |
| TAG 52:6 | 0.140 | 0.472 | 0.858 | 0.701 | 1.051 |
| TAG 54:2 | 0.355 | 0.628 | 0.911 | 0.747 | 1.111 |
| TAG 54:3 | 0.350 | 0.628 | 0.911 | 0.748 | 1.108 |
| TAG 54:4 | 0.310 | 0.581 | 0.904 | 0.745 | 1.098 |
| TAG 54:5 | 0.258 | 0.581 | 0.894 | 0.735 | 1.086 |
| TAG 54:6 | 0.131 | 0.471 | 0.858 | 0.704 | 1.046 |
| TAG 54:7 | 0.654 | 0.781 | 0.956 | 0.785 | 1.164 |
| TAG 56:5 | 0.976 | 0.818 | 1.003 | 0.825 | 1.219 |
| TAG 56:6 | 0.172 | 0.487 | 0.871 | 0.714 | 1.062 |
| TAG 56:7 | 0.593 | 0.763 | 0.948 | 0.778 | 1.154 |
| TAG 56:8 | 0.701 | 0.800 | 0.963 | 0.792 | 1.170 |
| TAG 58:7 | 0.130 | 0.471 | 0.836 | 0.664 | 1.054 |
| TAG 58:8 | 0.780 | 0.818 | 1.028 | 0.845 | 1.252 |
| TAG 58:9 | 0.527 | 0.717 | 1.070 | 0.868 | 1.319 |
| TAG 58:10 | 0.307 | 0.581 | 0.886 | 0.702 | 1.118 |

Values are odds ratios (95% confidence intervals) for cardiovascular disease from multivariate adjusted binary logistic regressions performed with the Z score of a given lipid specie obtained after log transformation. Chol, cholesterylester; DAG, diacylglyceride; LPC, lysophosphatidylcholine; PC, phosphatidyl-choline; PC-O, phosphatidylcholine ether; PE, phosphatidylethanolamine; PE-O, phosphatidylethanolamine ether; SM, sphingomyelin; TAG, triacylglyceride.
