# Supplementary material for: Plasma Lipid Composition and Risk of Developing Cardiovascular Disease
Source: PLoS One. 2013 Aug 15;8(8):e71846. doi: 10.1371/journal.pone.0071846 (PMC3744469; doi:10.1371/journal.pone.0071846)
Supplement: Table S4 — Relation of baseline triglycerides specie level to future adverse cardiovascular outcome adjusting for Framingham risk factors. (DOCX) [file pone.0071846.s007.docx]

**Supplementary Table S4.** Relation of baseline triglycerides specie level to future adverse cardiovascular outcome adjusting for Framingham risk factors

| Model | TAG48:1 (n=424) | TAG48:2 (n=424) | TAG48:3 (n=402) | TAG50:3 (n=424) | TAG50:4 (n=423) |
| --- | --- | --- | --- | --- | --- |
| Models adjusting for sex, age, BMI, type 2 diabetes, anti-hypertension treatment, smoking, LDL, HDL and SBP | | | | | |
| Lipid specie as categorical variable | | | | | |
| 1^st^ quartile | 1.0 (referent) | 1.0 (referent) | 1.0 (referent) | 1.0 (referent) | 1.0 (referent) |
| 2^nd^ quartile | 0.69 (0.40-1.20) | 0.76 (0.44-1.32) | 0.71 (0.40-1.25) | 0.90 (0.52-1.56) | 0.92 (0.53-1.58) |
| 3^rd^ quartile | 0.62 (0.35-1.10) | 0.59 (0.33-1.04) | 0.67 (0.37-1.19) | 0.93 (0.53-1.62) | 0.83 (0.47-1.45) |
| 4^th^ quartile | 0.67 (0.37-1.22) | 0.73 (0.41-1.31) | 0.64 (0.35-1.16) | 0.60 (0.33-1.11) | 0.63 (0.35-1.14) |
| *P* for trend | 0.165 | 0.195 | 0.142 | 0.145 | 0.125 |

Values are odds ratios (95% confidence intervals) for cardiovascular disease from multivariate adjusted binary logistic regressions performed with the Z score of a given triacylglyceride specie obtained after log transformation.

BMI, body mass index; HDL, high-density lipoprotein cholesterol; LDL, low-density lipoprotein cholesterol; SBP, systolic blood pressure; TAG, triacylglyceride.
