# Supplementary material for: Plasma Lipid Composition and Risk of Developing Cardiovascular Disease
Source: PLoS One. 2013 Aug 15;8(8):e71846. doi: 10.1371/journal.pone.0071846 (PMC3744469; doi:10.1371/journal.pone.0071846)
Supplement: Table S5 — Estimated q-values of the tests performed to study the association between CVD risk factors and the lipid species. (DOCX) [file pone.0071846.s008.docx]

**Supplementary Table S5.** Estimated q-values of the tests performed to study the association between CVD risk factors and the lipid species.

|  | SBP | | BMI | | HDL | | LDL | | Imtcca0 | | HbA1c | |
| --- | --- | --- | --- | --- | --- | --- | --- | --- | --- | --- | --- | --- |
|  | *P* | q-value | *P* | q-value | *P* | q-value | *P* | q-value | *P* | q-value | *P* | q-value |
| Chol16:1 | 1.50E-03 | 1.45E-03 | 6.11E-02 | 3.72E-02 | 3.25E-03 | 2.93E-03 | 1.13E-09 | 3.18E-08 | 1.74E-01 | 8.85E-02 | 5.21E-01 | 2.01E-01 |
| Chol16:0 | 2.62E-02 | 1.78E-02 | 5.14E-01 | 1.99E-01 | 1.50E-02 | 1.13E-02 | 8.93E-41 | 1.29E-37 | 1.52E-01 | 8.02E-02 | 3.80E-01 | 1.61E-01 |
| Chol18:1 | 5.14E-01 | 1.99E-01 | 2.53E-03 | 2.33E-03 | 5.31E-03 | 4.40E-03 | 1.37E-22 | 1.66E-20 | 8.16E-02 | 4.70E-02 | 5.01E-01 | 1.97E-01 |
| Chol18:2 | 3.54E-01 | 1.53E-01 | 5.72E-02 | 3.55E-02 | 1.19E-01 | 6.52E-02 | 6.12E-28 | 2.40E-25 | 2.39E-02 | 1.65E-02 | 6.56E-01 | 2.36E-01 |
| Chol18:3 | 6.34E-03 | 5.18E-03 | 5.35E-02 | 3.36E-02 | 5.88E-02 | 3.61E-02 | 2.27E-22 | 2.95E-20 | 3.05E-02 | 2.03E-02 | 9.53E-01 | 3.02E-01 |
| Chol20:3 | 1.95E-01 | 9.62E-02 | 6.84E-05 | 9.44E-05 | 9.54E-02 | 5.39E-02 | 1.73E-20 | 1.23E-18 | 8.09E-02 | 4.69E-02 | 5.59E-01 | 2.09E-01 |
| Chol20:4 | 4.80E-02 | 3.03E-02 | 1.88E-01 | 9.31E-02 | 1.11E-01 | 6.19E-02 | 9.93E-25 | 2.08E-22 | 7.00E-02 | 4.15E-02 | 9.41E-01 | 3.02E-01 |
| Chol20:5 | 1.18E-01 | 6.49E-02 | 8.01E-01 | 2.68E-01 | 3.38E-04 | 3.68E-04 | 4.92E-10 | 1.39E-08 | 4.76E-01 | 1.90E-01 | 5.26E-01 | 2.02E-01 |
| Chol22:6 | 1.82E-02 | 1.31E-02 | 7.49E-01 | 2.57E-01 | 8.39E-02 | 4.81E-02 | 1.29E-15 | 6.86E-14 | 4.60E-01 | 1.86E-01 | 2.60E-01 | 1.21E-01 |
| DAG36:2 | 1.59E-01 | 8.26E-02 | 5.15E-03 | 4.34E-03 | 1.08E-13 | 5.38E-12 | 8.54E-04 | 8.48E-04 | 1.38E-01 | 7.49E-02 | 2.01E-02 | 1.42E-02 |
| LPC16:0 | 2.45E-01 | 1.14E-01 | 3.67E-03 | 3.25E-03 | 1.45E-02 | 1.10E-02 | 1.54E-01 | 8.10E-02 | 9.42E-03 | 7.39E-03 | 1.16E-02 | 8.93E-03 |
| LPC18:1 | 1.76E-01 | 8.89E-02 | 2.39E-08 | 5.53E-07 | 4.80E-09 | 1.19E-07 | 7.17E-01 | 2.49E-01 | 3.85E-03 | 3.35E-03 | 2.41E-04 | 2.68E-04 |
| LPC18:3 | 1.49E-01 | 7.91E-02 | 2.66E-02 | 1.79E-02 | 3.90E-02 | 2.51E-02 | 1.78E-01 | 8.93E-02 | 3.24E-02 | 2.14E-02 | 7.79E-03 | 6.24E-03 |
| LPC18:0 | 3.54E-01 | 1.53E-01 | 5.28E-04 | 5.59E-04 | 1.75E-02 | 1.26E-02 | 1.97E-03 | 1.87E-03 | 2.79E-02 | 1.87E-02 | 3.48E-03 | 3.09E-03 |
| LPC20:4 | 3.92E-02 | 2.52E-02 | 1.96E-04 | 2.24E-04 | 5.50E-04 | 5.77E-04 | 3.56E-01 | 1.53E-01 | 5.91E-01 | 2.18E-01 | 7.39E-02 | 4.34E-02 |
| PC32:1 | 2.19E-03 | 2.04E-03 | 5.54E-03 | 4.57E-03 | 1.87E-04 | 2.19E-04 | 5.20E-03 | 4.36E-03 | 9.48E-01 | 3.02E-01 | 7.70E-01 | 2.62E-01 |
| PC32:0 | 2.24E-02 | 1.56E-02 | 5.99E-01 | 2.19E-01 | 2.72E-06 | 3.49E-05 | 8.34E-14 | 4.59E-12 | 8.89E-01 | 2.91E-01 | 3.79E-01 | 1.61E-01 |
| PC34:1 | 1.86E-01 | 9.27E-02 | 5.01E-02 | 3.16E-02 | 7.12E-08 | 1.80E-06 | 2.96E-05 | 4.76E-05 | 9.70E-01 | 3.05E-01 | 9.67E-01 | 3.05E-01 |
| PC34:2 | 6.61E-01 | 2.37E-01 | 5.86E-02 | 3.61E-02 | 2.65E-05 | 4.36E-05 | 9.05E-13 | 4.00E-11 | 5.90E-01 | 2.18E-01 | 1.68E-01 | 8.58E-02 |
| PC34:3 | 3.98E-01 | 1.67E-01 | 2.63E-01 | 1.21E-01 | 1.12E-05 | 2.12E-05 | 2.37E-06 | 3.49E-05 | 5.11E-01 | 1.99E-01 | 6.97E-01 | 2.45E-01 |
| PC36:2 | 9.69E-01 | 3.05E-01 | 1.88E-01 | 9.31E-02 | 2.34E-04 | 2.63E-04 | 9.20E-14 | 4.98E-12 | 7.95E-01 | 2.67E-01 | 6.45E-01 | 2.34E-01 |
| PC36:3 | 9.17E-01 | 2.97E-01 | 1.36E-01 | 7.40E-02 | 3.26E-03 | 2.93E-03 | 6.15E-07 | 1.34E-05 | 8.75E-01 | 2.88E-01 | 7.75E-01 | 2.63E-01 |
| PC36:4 | 8.12E-02 | 4.69E-02 | 5.12E-01 | 1.99E-01 | 1.18E-06 | 2.07E-05 | 1.74E-07 | 2.60E-06 | 4.80E-01 | 1.91E-01 | 4.04E-01 | 1.69E-01 |
| PC36:5 | 6.73E-02 | 4.02E-02 | 6.94E-01 | 2.45E-01 | 1.71E-07 | 2.60E-06 | 1.94E-04 | 2.23E-04 | 4.48E-01 | 1.83E-01 | 3.03E-01 | 1.35E-01 |
| PC38:3 | 3.91E-01 | 1.64E-01 | 1.08E-05 | 2.10E-05 | 6.93E-01 | 2.45E-01 | 2.90E-09 | 6.19E-08 | 4.76E-01 | 1.90E-01 | 6.95E-01 | 2.45E-01 |
| PC38:4 | 4.69E-01 | 1.88E-01 | 3.72E-02 | 2.41E-02 | 7.21E-03 | 5.83E-03 | 5.65E-10 | 1.69E-08 | 7.68E-01 | 2.62E-01 | 8.74E-01 | 2.88E-01 |
| PC38:5 | 9.27E-02 | 5.26E-02 | 6.04E-01 | 2.20E-01 | 2.84E-08 | 5.56E-07 | 8.53E-07 | 1.75E-05 | 5.76E-01 | 2.14E-01 | 8.83E-01 | 2.90E-01 |
| PC38:6 | 1.42E-01 | 7.60E-02 | 2.94E-01 | 1.32E-01 | 1.04E-05 | 2.07E-05 | 8.91E-05 | 1.16E-04 | 5.55E-02 | 3.46E-02 | 6.54E-01 | 2.36E-01 |
| PC38:7 | 9.58E-02 | 5.40E-02 | 2.35E-01 | 1.11E-01 | 1.06E-02 | 8.26E-03 | 2.16E-03 | 2.02E-03 | 4.85E-01 | 1.92E-01 | 5.52E-01 | 2.07E-01 |
| PC40:6 | 7.36E-02 | 4.34E-02 | 2.29E-01 | 1.09E-01 | 2.11E-02 | 1.47E-02 | 2.45E-06 | 3.49E-05 | 3.80E-01 | 1.61E-01 | 3.51E-01 | 1.53E-01 |
| PC40:7 | 2.41E-01 | 1.13E-01 | 8.29E-01 | 2.75E-01 | 3.83E-03 | 3.35E-03 | 9.09E-07 | 1.90E-05 | 5.50E-01 | 2.07E-01 | 6.13E-02 | 3.72E-02 |
| PC40:8 | 7.98E-01 | 2.67E-01 | 6.05E-01 | 2.20E-01 | 2.99E-05 | 4.76E-05 | 1.25E-05 | 2.22E-05 | 5.73E-01 | 2.13E-01 | 2.72E-01 | 1.25E-01 |
| PCO34:2 | 4.77E-01 | 1.90E-01 | 8.04E-05 | 1.08E-04 | 2.57E-12 | 8.66E-11 | 1.51E-02 | 1.13E-02 | 4.91E-01 | 1.94E-01 | 2.04E-02 | 1.44E-02 |
| PCO34:3 | 4.33E-01 | 1.78E-01 | 3.59E-04 | 3.88E-04 | 6.37E-21 | 7.43E-19 | 1.14E-04 | 1.40E-04 | 2.02E-01 | 9.87E-02 | 4.40E-03 | 3.72E-03 |
| PCO36:4 | 6.77E-01 | 2.41E-01 | 2.78E-01 | 1.27E-01 | 5.74E-06 | 7.12E-05 | 2.00E-06 | 2.10E-05 | 2.40E-01 | 1.13E-01 | 1.25E-01 | 6.86E-02 |
| PCO36:5 | 5.30E-01 | 2.03E-01 | 3.40E-01 | 1.50E-01 | 2.82E-13 | 1.07E-11 | 1.09E-06 | 2.07E-05 | 2.45E-01 | 1.14E-01 | 6.05E-01 | 2.20E-01 |
| PCO38:4 | 2.81E-01 | 1.28E-01 | 3.55E-02 | 2.34E-02 | 3.96E-02 | 2.53E-02 | 1.67E-04 | 2.00E-04 | 3.43E-01 | 1.50E-01 | 2.51E-01 | 1.17E-01 |
| PCO38:5 | 5.70E-01 | 2.13E-01 | 1.50E-02 | 1.13E-02 | 9.49E-05 | 1.20E-04 | 4.10E-03 | 3.53E-03 | 7.55E-01 | 2.59E-01 | 6.90E-01 | 2.45E-01 |
| PCO38:6 | 1.83E-01 | 9.15E-02 | 5.13E-01 | 1.99E-01 | 8.76E-07 | 1.75E-05 | 5.85E-04 | 6.07E-04 | 9.51E-01 | 3.02E-01 | 3.03E-02 | 2.03E-02 |
| PE36:2 | 9.77E-01 | 3.07E-01 | 2.58E-01 | 1.20E-01 | 5.96E-01 | 2.19E-01 | 5.02E-07 | 1.13E-05 | 8.95E-01 | 2.92E-01 | 1.58E-01 | 8.22E-02 |
| PE38:2 | 1.77E-01 | 8.93E-02 | 1.02E-04 | 1.26E-04 | 3.62E-06 | 4.76E-05 | 4.20E-13 | 1.92E-11 | 5.88E-02 | 3.61E-02 | 2.67E-01 | 1.23E-01 |
| PE38:4 | 6.74E-01 | 2.41E-01 | 8.83E-02 | 5.05E-02 | 7.60E-01 | 2.60E-01 | 1.65E-01 | 8.50E-02 | 6.97E-01 | 2.45E-01 | 1.42E-01 | 7.60E-02 |
| PEO38:6 | 7.74E-02 | 4.54E-02 | 3.85E-01 | 1.63E-01 | 4.46E-05 | 6.48E-05 | 2.27E-03 | 2.10E-03 | 7.28E-01 | 2.51E-01 | 2.02E-01 | 9.87E-02 |
| SM32:1 | 9.82E-01 | 3.08E-01 | 3.54E-01 | 1.53E-01 | 1.76E-04 | 2.09E-04 | 2.41E-14 | 1.21E-12 | 9.31E-01 | 3.00E-01 | 7.19E-01 | 2.49E-01 |
| SM34:1 | 4.58E-01 | 1.86E-01 | 4.63E-06 | 5.96E-05 | 9.66E-09 | 2.55E-07 | 1.45E-25 | 2.79E-23 | 8.11E-01 | 2.70E-01 | 6.01E-01 | 2.20E-01 |
| SM34:2 | 7.68E-01 | 2.62E-01 | 9.64E-01 | 3.05E-01 | 1.55E-06 | 2.08E-05 | 2.17E-18 | 2.02E-16 | 4.62E-01 | 1.86E-01 | 1.67E-01 | 8.56E-02 |
| SM36:1 | 9.17E-01 | 2.97E-01 | 8.92E-01 | 2.92E-01 | 2.11E-02 | 1.47E-02 | 1.45E-17 | 8.91E-16 | 5.06E-01 | 1.98E-01 | 7.90E-01 | 2.66E-01 |
| SM38:1 | 7.95E-01 | 2.67E-01 | 2.93E-01 | 1.32E-01 | 6.07E-02 | 3.70E-02 | 2.10E-29 | 1.07E-26 | 4.48E-01 | 1.83E-01 | 8.40E-01 | 2.78E-01 |
| SM38:2 | 7.10E-01 | 2.47E-01 | 4.38E-01 | 1.80E-01 | 2.62E-01 | 1.21E-01 | 7.36E-11 | 2.56E-09 | 1.12E-01 | 6.24E-02 | 3.85E-01 | 1.63E-01 |
| SM40:1 | 7.06E-01 | 2.47E-01 | 1.56E-01 | 8.17E-02 | 5.55E-02 | 3.46E-02 | 3.49E-37 | 2.44E-34 | 2.40E-02 | 1.65E-02 | 2.94E-01 | 1.32E-01 |
| SM40:2 | 3.58E-01 | 1.53E-01 | 2.59E-02 | 1.77E-02 | 1.88E-04 | 2.19E-04 | 1.73E-27 | 3.43E-25 | 4.79E-01 | 1.90E-01 | 5.02E-01 | 1.97E-01 |
| SM41:1 | 4.56E-01 | 1.85E-01 | 2.04E-01 | 9.91E-02 | 9.96E-02 | 5.59E-02 | 3.80E-24 | 6.16E-22 | 7.97E-02 | 4.64E-02 | 9.30E-01 | 3.00E-01 |
| SM42:1 | 2.17E-01 | 1.04E-01 | 4.21E-02 | 2.68E-02 | 6.39E-03 | 5.19E-03 | 1.67E-12 | 4.61E-11 | 5.45E-01 | 2.07E-01 | 2.99E-01 | 1.34E-01 |
| SM42:2 | 9.33E-01 | 3.00E-01 | 6.89E-04 | 6.93E-04 | 9.91E-09 | 2.55E-07 | 5.42E-17 | 3.67E-15 | 5.98E-01 | 2.19E-01 | 1.94E-01 | 9.59E-02 |
| SM42:3 | 3.93E-01 | 1.65E-01 | 2.62E-02 | 1.78E-02 | 5.20E-12 | 1.96E-10 | 4.42E-12 | 1.62E-10 | 3.40E-01 | 1.50E-01 | 1.08E-04 | 1.33E-04 |
| TAG46:1 | 1.84E-02 | 1.32E-02 | 1.42E-05 | 2.50E-05 | 9.38E-06 | 1.16E-04 | 3.24E-02 | 2.14E-02 | 6.66E-01 | 2.38E-01 | 1.57E-02 | 1.16E-02 |
| TAG46:2 | 6.27E-02 | 3.79E-02 | 9.79E-04 | 9.61E-04 | 2.10E-06 | 4.94E-06 | 6.67E-02 | 4.00E-02 | 4.50E-01 | 1.83E-01 | 1.15E-01 | 6.35E-02 |
| TAG48:1 | 1.58E-03 | 1.51E-03 | 2.84E-09 | 6.19E-08 | 2.22E-09 | 6.19E-08 | 2.47E-04 | 2.72E-04 | 1.41E-01 | 7.57E-02 | 3.42E-03 | 3.06E-03 |
| TAG48:2 | 1.49E-02 | 1.13E-02 | 3.78E-07 | 7.14E-06 | 4.84E-10 | 1.39E-08 | 2.84E-04 | 3.11E-04 | 1.76E-01 | 8.89E-02 | 4.27E-02 | 2.71E-02 |
| TAG48:3 | 1.58E-01 | 8.22E-02 | 6.25E-04 | 6.41E-04 | 1.46E-08 | 2.89E-07 | 1.68E-02 | 1.23E-02 | 4.07E-01 | 1.69E-01 | 3.56E-01 | 1.53E-01 |
| TAG50:1 | 8.72E-04 | 8.61E-04 | 7.33E-11 | 2.56E-09 | 1.17E-11 | 3.87E-10 | 8.39E-05 | 1.11E-04 | 1.66E-01 | 8.56E-02 | 2.53E-06 | 3.49E-05 |
| TAG50:2 | 8.49E-03 | 6.73E-03 | 7.07E-10 | 2.24E-08 | 3.97E-13 | 1.55E-11 | 9.51E-05 | 1.20E-04 | 2.04E-01 | 9.90E-02 | 1.26E-03 | 1.22E-03 |
| TAG50:3 | 8.99E-02 | 5.12E-02 | 7.78E-08 | 1.80E-06 | 2.59E-15 | 1.32E-13 | 3.55E-05 | 5.31E-05 | 3.57E-01 | 1.53E-01 | 3.57E-02 | 2.34E-02 |
| TAG50:4 | 1.39E-01 | 7.49E-02 | 1.10E-05 | 2.10E-05 | 3.90E-12 | 1.27E-10 | 2.21E-04 | 2.51E-04 | 3.23E-01 | 1.43E-01 | 1.70E-01 | 8.64E-02 |
| TAG51:2 | 1.61E-01 | 8.34E-02 | 1.32E-06 | 2.07E-05 | 4.81E-13 | 1.92E-11 | 7.23E-08 | 1.80E-06 | 2.11E-01 | 1.02E-01 | 8.48E-03 | 6.73E-03 |
| TAG51:3 | 5.49E-01 | 2.07E-01 | 6.04E-04 | 6.24E-04 | 1.03E-16 | 6.98E-15 | 1.25E-05 | 2.22E-05 | 5.45E-01 | 2.07E-01 | 1.97E-01 | 9.66E-02 |
| TAG52:2 | 1.02E-01 | 5.70E-02 | 1.00E-07 | 2.50E-06 | 5.18E-17 | 3.67E-15 | 1.15E-05 | 2.14E-05 | 7.83E-02 | 4.57E-02 | 6.74E-05 | 9.37E-05 |
| TAG52:3 | 5.45E-01 | 2.07E-01 | 2.94E-06 | 3.49E-05 | 2.15E-23 | 3.58E-21 | 9.06E-07 | 1.90E-05 | 2.18E-01 | 1.04E-01 | 6.60E-04 | 6.72E-04 |
| TAG52:4 | 8.37E-01 | 2.77E-01 | 3.22E-05 | 4.91E-05 | 5.13E-21 | 6.68E-19 | 8.89E-06 | 1.08E-04 | 4.31E-01 | 1.78E-01 | 4.29E-03 | 3.67E-03 |
| TAG52:5 | 5.27E-01 | 2.02E-01 | 4.68E-05 | 6.68E-05 | 4.98E-17 | 3.28E-15 | 4.56E-05 | 6.57E-05 | 5.94E-01 | 2.19E-01 | 1.80E-02 | 1.30E-02 |
| TAG52:6 | 5.22E-01 | 2.01E-01 | 4.51E-06 | 1.03E-05 | 3.04E-06 | 4.76E-05 | 1.70E-02 | 1.24E-02 | 5.46E-01 | 2.07E-01 | 1.27E-01 | 6.95E-02 |
| TAG54:2 | 1.51E-01 | 8.00E-02 | 7.76E-06 | 9.68E-05 | 3.70E-15 | 1.89E-13 | 3.27E-06 | 4.76E-05 | 2.30E-02 | 1.59E-02 | 1.21E-05 | 2.19E-05 |
| TAG54:3 | 4.05E-01 | 1.69E-01 | 6.73E-04 | 6.82E-04 | 1.58E-17 | 8.91E-16 | 1.28E-04 | 1.54E-04 | 6.34E-02 | 3.81E-02 | 9.89E-05 | 1.24E-04 |
| TAG54:4 | 9.44E-01 | 3.02E-01 | 9.29E-03 | 7.32E-03 | 4.35E-19 | 4.30E-17 | 1.89E-04 | 2.19E-04 | 2.95E-01 | 1.32E-01 | 1.78E-03 | 1.70E-03 |
| TAG54:5 | 9.12E-01 | 2.96E-01 | 2.00E-03 | 1.88E-03 | 8.98E-18 | 7.62E-16 | 1.28E-04 | 1.54E-04 | 5.60E-01 | 2.09E-01 | 2.91E-03 | 2.66E-03 |
| TAG54:6 | 2.35E-01 | 1.11E-01 | 1.19E-05 | 2.19E-05 | 6.95E-13 | 2.79E-11 | 5.17E-04 | 5.51E-04 | 8.21E-01 | 2.73E-01 | 7.19E-04 | 7.19E-04 |
| TAG54:7 | 1.43E-01 | 7.63E-02 | 3.08E-05 | 4.76E-05 | 3.09E-05 | 4.76E-05 | 6.00E-03 | 4.92E-03 | 8.98E-01 | 2.93E-01 | 1.31E-02 | 1.01E-02 |
| TAG56:5 | 5.40E-01 | 2.06E-01 | 2.39E-04 | 2.68E-04 | 1.18E-10 | 3.62E-09 | 4.02E-06 | 5.93E-05 | 4.31E-01 | 1.78E-01 | 5.52E-04 | 5.77E-04 |
| TAG56:6 | 2.11E-01 | 1.02E-01 | 1.09E-03 | 1.06E-03 | 7.05E-10 | 2.24E-08 | 2.80E-07 | 4.94E-06 | 2.93E-01 | 1.32E-01 | 8.71E-05 | 1.14E-04 |
| TAG56:7 | 3.41E-01 | 1.50E-01 | 3.50E-05 | 5.28E-05 | 5.54E-06 | 7.12E-05 | 3.81E-03 | 3.35E-03 | 9.01E-01 | 2.93E-01 | 5.02E-04 | 5.39E-04 |
| TAG56:8 | 7.32E-01 | 2.52E-01 | 7.51E-05 | 1.02E-04 | 9.04E-05 | 1.16E-04 | 9.63E-03 | 7.51E-03 | 7.19E-01 | 2.49E-01 | 1.41E-02 | 1.08E-02 |
| TAG58:7 | 3.01E-01 | 1.34E-01 | 3.93E-03 | 3.40E-03 | 7.76E-03 | 6.24E-03 | 4.24E-01 | 1.76E-01 | 6.51E-01 | 2.35E-01 | 1.16E-02 | 8.93E-03 |
| TAG58:8 | 7.03E-01 | 2.47E-01 | 4.31E-03 | 3.67E-03 | 3.15E-03 | 2.87E-03 | 6.78E-02 | 4.03E-02 | 9.48E-01 | 3.02E-01 | 3.62E-02 | 2.37E-02 |
| TAG58:9 | 7.10E-01 | 2.47E-01 | 1.58E-02 | 1.17E-02 | 1.73E-02 | 1.26E-02 | 7.27E-01 | 2.51E-01 | 7.84E-01 | 2.65E-01 | 5.27E-03 | 4.40E-03 |
| TAG58:10 | 7.90E-01 | 2.66E-01 | 3.69E-02 | 2.40E-02 | 3.23E-01 | 1.43E-01 | 2.20E-01 | 1.05E-01 | 3.64E-01 | 1.56E-01 | 1.87E-02 | 1.33E-02 |

Partial correlations were performed between the lipid species after log transformation and current known laboratory predictors for cardiovascular disease, adjusting for age and sex. Q-values were calculated using the QVALUE software. BMI, body mass index; Chol, cholesterylester; DAG, diacylglyceride; HbA1c, haemoglobin A1c; HDL, high-density lipoprotein cholesterol; Imtcca0, intima-media thickness of the common carotid artery at baseline; LDL, low-density lipoprotein cholesterol; LPC, lysophosphatidylcholine; PC, phosphatidyl-choline; PC-O, phosphatidylcholine ether; PE, phosphatidylethanolamine; PE-O, phosphatidylethanolamine ether; SBP, systolic blood pressure; SM, sphingomyelin; TAG, triacylglyceride.
