# Supplementary material for: Plasma Lipid Composition and Risk of Developing Cardiovascular Disease
Source: PLoS One. 2013 Aug 15;8(8):e71846. doi: 10.1371/journal.pone.0071846 (PMC3744469; doi:10.1371/journal.pone.0071846)
Supplement: Table S6 — Relation between 23 validated coronary artery disease associated gene variants and baseline plasma lipid metabolites level. (DOCX) [file pone.0071846.s009.docx]

**Supplementary Table S6.** Relation between 23 validated coronary artery disease associated gene variants and baseline plasma lipid metabolites level.

| SNP | Gene(s) in region | Number of associated lipid species |
| --- | --- | --- |
| rs6725887 | WDR12 | 21 |
| rs17114036 | PPAP2B | 20 |
| rs3798220 | LPA | 17 |
| rs646776 | SORT1 | 10 |
| rs11556924 | ZC3HC1 | 7 |
| rs12936587 | PEMT, RASD1, SMCR3 | 6 |
| rs9349379 | PHACTR1 | 5 |
| rs964184 | ZNF259, APOA5-A4-C3-A1 | 5 |
| rs12413409 | CNNM2, CYP17A1, NT5C2 | 4 |
| rs2895811 | HHIPL1, KIAA1822 | 4 |
| rs17609940 | ANKS1A | 3 |
| rs12190287 | TCF21 | 3 |
| rs9982601 | MRPS6, KCNE2 | 3 |
| rs216172 | SMG6, SRR | 2 |
| rs1746048 | CXCL12 | 2 |
| rs9818870 | MRAS | 2 |
| rs9411489 | ABO | 1 |
| rs3184504 | SH2B3 | 1 |
| rs6511720 | LDLR | 0 |
| rs3825807 | ADAMTS7 | 0 |
| rs11206510 | PCSK9 | 0 |
| rs17465637 | MIA3 | 0 |
| rs4977574 | CDKN2A, CDKN2B | 0 |

Linear regressions were performed between the CAD-associated locus and the lipid species after log transformation adjusting for age and sex.
