# Supplementary material for: Plasma Lipid Composition and Risk of Developing Cardiovascular Disease
Source: PLoS One. 2013 Aug 15;8(8):e71846. doi: 10.1371/journal.pone.0071846 (PMC3744469; doi:10.1371/journal.pone.0071846)
Supplement: Table S7 — The risk allele of 8 of the validated coronary artery disease associated gene variants shows significant association with the baseline plasma level of several lipid species. (DOCX) [file pone.0071846.s010.docx]

**Supplementary Table S7.** The risk allele of 8 of the validated coronary artery disease associated gene variants shows significant association with the baseline plasma level of several lipid species.

|  | rs6725887_WDR12 | | rs17114036_PPAP2B | | rs3798220_ LPA | | rs646776_ SORT1 | | rs11556924_ZC3HC1 | | rs12936587_PEMT, RASD1, SMCR3 | | rs9349379_PHACTR1 | | rs964184_ ZNF259,APOA5-A4-C3-A1 | |
| --- | --- | --- | --- | --- | --- | --- | --- | --- | --- | --- | --- | --- | --- | --- | --- | --- |
| Lipid specie | β (SE) | *P* | β (SE) | *P* | β (SE) | *P* | β (SE) | *P* | β (SE) | *P* | β (SE) | *P* | β (SE) | *P* | β (SE) | *P* |
| Chol16:0 | **-0.069 (0.034)** | **4.50E-02** | -0.049 (0.042) | 2.38E-01 | 0.110 (0.098) | 2.63E-01 | 0.010 (0.029) | 7.17E-01 | 0.024 (0.023) | 3.12E-01 | -0.014 (0.023) | 5.52E-01 | 0.028 (0.023) | 2.29E-01 | 0.016 (0.033) | 6.38E-01 |
| Chol16:1 | -0.061 (0.052) | 2.47E-01 | **-0.149 (0.062)** | **1.72E-02** | 0.260 (0.149) | 8.19E-02 | 0.057 (0.044) | 1.91E-01 | 0.057 (0.035) | 1.10E-01 | 0.036 (0.035) | 3.04E-01 | -0.015 (0.035) | 6.62E-01 | -0.071 (0.051) | 1.60E-01 |
| Chol18:1 | -0.092 (0.050) | 6.56E-02 | **-0.138 (0.061)** | **2.50E-02** | 0.113 (0.146) | 4.40E-01 | 0.008 (0.043) | 8.58E-01 | **0.067 (0.034)** | **5.04E-02** | 0.026 (0.033) | 4.33E-01 | 0.005 (0.034) | 8.76E-01 | -0.072 (0.048) | 1.36E-01 |
| Chol18:2 | -0.028 (0.039) | 4.79E-01 | 0.060 (0.047) | 2.04E-01 | -0.204 (0.112) | 6.94E-02 | -0.001 (0.033) | 9.67E-01 | 0.003 (0.027) | 9.23E-01 | -0.023 (0.026) | 3.65E-01 | 0.026 (0.026) | 3.23E-01 | 0.037 (0.038) | 3.27E-01 |
| Chol18:3 | **-0.086 (0.040)** | **3.28E-02** | -0.049 (0.049) | 3.21E-01 | 0.011 (0.116) | 9.26E-01 | 0.018 (0.034) | 6.01E-01 | 0.049 (0.028) | 7.48E-02 | -0.016 (0.027) | 5.42E-01 | -0.026 (0.027) | 3.27E-01 | 0.029 (0.040) | 4.72E-01 |
| Chol20:3 | -0.039 (0.050) | 4.36E-01 | **-0.159 (0.062)** | **1.08E-02** | 0.037 (0.147) | 8.00E-01 | 0.038 (0.042) | 3.62E-01 | 0.041 (0.035) | 2.33E-01 | -0.022 (0.033) | 5.18E-01 | -0.031 (0.034) | 3.56E-01 | -0.022 (0.050) | 6.51E-01 |
| Chol20:4 | -0.066 (0.040) | 9.97E-02 | -0.039 (0.048) | 4.18E-01 | -0.017 (0.114) | 8.82E-01 | 0.022 (0.033) | 5.03E-01 | 0.052 (0.027) | 5.44E-02 | 0.002 (0.026) | 9.48E-01 | -0.005 (0.026) | 8.50E-01 | -0.009 (0.039) | 8.22E-01 |
| Chol20:5 | -0.078 (0.068) | 2.53E-01 | -0.010 (0.083) | 9.03E-01 | 0.110 (0.194) | 5.72E-01 | -0.092 (0.055) | 9.82E-02 | 0.051 (0.046) | 2.62E-01 | -0.037 (0.045) | 4.08E-01 | 0.022 (0.046) | 6.26E-01 | 0.083 (0.066) | 2.07E-01 |
| Chol22:6 | -0.050 (0.048) | 2.98E-01 | -0.022 (0.058) | 7.00E-01 | -0.096 (0.136) | 4.80E-01 | -0.062 (0.039) | 1.11E-01 | 0.035 (0.032) | 2.83E-01 | -0.014 (0.032) | 6.51E-01 | 0.057 (0.032) | 6.94E-02 | 0.060 (0.046) | 1.96E-01 |
| DAG36:2 | -0.094 (0.073) | 1.97E-01 | 0.056 (0.086) | 5.14E-01 | 0.229 (0.193) | 2.37E-01 | 0.067 (0.061) | 2.69E-01 | -0.055 (0.048) | 2.52E-01 | 0.056 (0.046) | 2.32E-01 | 0.064 (0.047) | 1.80E-01 | 0.086 (0.070) | 2.18E-01 |
| LPC16:0 | -0.076 (0.049) | 1.17E-01 | **-0.113 (0.059)** | **5.56E-02** | 0.192 (0.138) | 1.67E-01 | -0.027 (0.041) | 5.17E-01 | -0.018 (0.033) | 5.94E-01 | **-0.070 (0.032)** | **3.09E-02** | 0.011 (0.033) | 7.37E-01 | -0.052 (0.047) | 2.70E-01 |
| LPC18:0 | **-0.090 (0.044)** | **4.22E-02** | -0.042 (0.053) | 4.32E-01 | 0.084 (0.126) | 5.06E-01 | -0.035 (0.037) | 3.44E-01 | -0.043 (0.030) | 1.58E-01 | -0.056 (0.029) | 5.63E-02 | 0.010 (0.029) | 7.32E-01 | -0.003 (0.043) | 9.53E-01 |
| LPC18:1 | -0.087 (0.046) | 5.90E-02 | **-0.121 (0.056)** | **3.10E-02** | 0.086 (0.132) | 5.15E-01 | -0.063 (0.039) | 1.13E-01 | -0.011 (0.032) | 7.33E-01 | -0.039 (0.031) | 2.06E-01 | 0.009 (0.030) | 7.76E-01 | -0.065 (0.045) | 1.49E-01 |
| LPC18:3 | **-0.146 (0.061)** | **1.76E-02** | -0.053 (0.075) | 4.79E-01 | 0.031 (0.175) | 8.60E-01 | -0.085 (0.050) | 9.23E-02 | -0.037 (0.042) | 3.74E-01 | -0.067 (0.041) | 1.04E-01 | -0.015 (0.041) | 7.11E-01 | -0.072 (0.060) | 2.35E-01 |
| LPC20:4 | -0.010 (0.074) | 8.95E-01 | -0.078 (0.085) | 3.64E-01 | -0.043 (0.215) | 8.42E-01 | **-0.157 (0.062)** | **1.16E-02** | -0.053 (0.050) | 2.87E-01 | **-0.100 (0.050)** | **4.65E-02** | 0.036 (0.050) | 4.65E-01 | -0.021 (0.075) | 7.77E-01 |
| PC32:0 | -0.056 (0.031) | 6.75E-02 | **-0.073 (0.037)** | **5.30E-02** | 0.166 (0.087) | 5.87E-02 | 0.006 (0.026) | 8.28E-01 | 0.028 (0.021) | 1.72E-01 | -0.012 (0.020) | 5.49E-01 | 0 (0.021) | 9.86E-01 | -0.016 (0.030) | 5.98E-01 |
| PC32:1 | -0.063 (0.058) | 2.78E-01 | **-0.172 (0.070)** | **1.41E-02** | 0.321 (0.166) | 5.36E-02 | 0.083 (0.048) | 8.80E-02 | **0.087 (0.039)** | **2.63E-02** | 0.041 (0.039) | 2.89E-01 | -0.041 (0.039) | 2.94E-01 | -0.085 (0.057) | 1.37E-01 |
| PC34:1 | -0.088 (0.048) | 6.43E-02 | **-0.157 (0.058)** | **7.04E-03** | **0.351 (0.137)** | **1.08E-02** | 0.015 (0.041) | 7.07E-01 | 0.061 (0.033) | 6.19E-02 | 0.009 (0.032) | 7.66E-01 | 0.015 (0.032) | 6.49E-01 | -0.070 (0.046) | 1.33E-01 |
| PC34:2 | -0.012 (0.038) | 7.60E-01 | -0.039 (0.047) | 4.01E-01 | 0.115 (0.110) | 2.98E-01 | 0.024 (0.032) | 4.56E-01 | 0.011 (0.026) | 6.66E-01 | -0.027 (0.025) | 2.85E-01 | 0.022 (0.026) | 3.90E-01 | 0.015 (0.037) | 6.89E-01 |
| PC34:3 | **-0.089 (0.039)** | **2.33E-02** | -0.070 (0.048) | 1.46E-01 | 0.119 (0.115) | 3.01E-01 | 0.018 (0.033) | 5.78E-01 | 0.047 (0.027) | 7.99E-02 | -0.011 (0.026) | 6.66E-01 | -0.009 (0.027) | 7.37E-01 | -0.071 (0.038) | 6.51E-02 |
| PC36:2 | -0.025 (0.039) | 5.19E-01 | -0.057 (0.047) | 2.28E-01 | 0.196 (0.111) | 7.80E-02 | 0.016 (0.033) | 6.25E-01 | 0.003 (0.027) | 9.17E-01 | -0.028 (0.026) | 2.70E-01 | 0.014 (0.026) | 5.94E-01 | 0.007 (0.038) | 8.48E-01 |
| PC36:3 | **-0.095 (0.043)** | **2.82E-02** | **-0.133 (0.053)** | **1.25E-02** | 0.196 (0.125) | 1.18E-01 | 0.017 (0.036) | 6.36E-01 | 0.047 (0.030) | 1.10E-01 | -0.033 (0.029) | 2.53E-01 | 0 (0.029) | 9.89E-01 | -0.037 (0.042) | 3.83E-01 |
| PC36:4 | **-0.086 (0.039)** | **2.86E-02** | **-0.119 (0.047)** | **1.20E-02** | 0.138 (0.113) | 2.22E-01 | 0.025 (0.033) | 4.51E-01 | **0.054 (0.027)** | **4.51E-02** | -0.016 (0.026) | 5.33E-01 | -0.008 (0.026) | 7.50E-01 | -0.003 (0.038) | 9.45E-01 |
| PC36:5 | -0.083 (0.050) | 9.75E-02 | -0.077 (0.061) | 2.04E-01 | 0.110 (0.145) | 4.48E-01 | -0.042 (0.041) | 3.14E-01 | 0.038 (0.034) | 2.63E-01 | **-0.069 (0.033)** | **3.74E-02** | 0.026 (0.033) | 4.31E-01 | 0.006 (0.049) | 9.07E-01 |
| PC38:3 | -0.056 (0.042) | 1.75E-01 | **-0.149 (0.050)** | **3.34E-03** | 0.224 (0.120) | 6.27E-02 | 0.046 (0.035) | 1.87E-01 | 0.007 (0.028) | 8.15E-01 | -0.022 (0.028) | 4.24E-01 | -0.033 (0.028) | 2.44E-01 | -0.010 (0.041) | 8.02E-01 |
| PC38:4 | **-0.085 (0.036)** | **1.88E-02** | **-0.123 (0.043)** | **4.65E-03** | 0.165 (0.104) | 1.16E-01 | 0.012 (0.030) | 6.84E-01 | 0.035 (0.025) | 1.64E-01 | -0.027 (0.024) | 2.65E-01 | -0.014 (0.024) | 5.69E-01 | -0.004 (0.035) | 9.03E-01 |
| PC38:5 | **-0.108 (0.040)** | **7.01E-03** | **-0.127 (0.048)** | **8.81E-03** | 0.181 (0.116) | 1.21E-01 | -0.021 (0.034) | 5.27E-01 | 0.040 (0.028) | 1.42E-01 | **-0.054 (0.027)** | **4.32E-02** | 0.014 (0.027) | 6.14E-01 | 0.016 (0.039) | 6.74E-01 |
| PC38:6 | -0.079 (0.045) | 7.58E-02 | **-0.114 (0.054)** | **3.58E-02** | 0.101 (0.129) | 4.35E-01 | -0.065 (0.037) | 8.17E-02 | 0.030 (0.031) | 3.21E-01 | -0.044 (0.030) | 1.41E-01 | 0.050 (0.030) | 9.70E-02 | 0.058 (0.044) | 1.85E-01 |
| PC38:7 | -0.050 (0.057) | 3.72E-01 | -0.058 (0.069) | 4.06E-01 | -0.020 (0.164) | 9.04E-01 | -0.034 (0.048) | 4.80E-01 | **0.077 (0.039)** | **4.94E-02** | 0.003 (0.038) | 9.41E-01 | -0.006 (0.038) | 8.83E-01 | -0.031 (0.056) | 5.83E-01 |
| PC40:6 | -0.069 (0.041) | 9.42E-02 | **-0.113 (0.050)** | **2.32E-02** | 0.195 (0.118) | 9.89E-02 | -0.051 (0.034) | 1.37E-01 | 0.009 (0.028) | 7.41E-01 | -0.025 (0.027) | 3.52E-01 | 0.029 (0.028) | 2.88E-01 | 0.031 (0.040) | 4.38E-01 |
| PC40:7 | **-0.100 (0.037)** | **7.16E-03** | -0.063 (0.045) | 1.59E-01 | 0.011 (0.108) | 9.18E-01 | -0.030 (0.031) | 3.44E-01 | 0.019 (0.026) | 4.65E-01 | -0.026 (0.025) | 2.95E-01 | 0.008 (0.025) | 7.48E-01 | 0.026 (0.036) | 4.81E-01 |
| PC40:8 | **-0.126 (0.056)** | **2.47E-02** | -0.126 (0.067) | 6.08E-02 | -0.028 (0.167) | 8.66E-01 | **-0.095 (0.047)** | **4.20E-02** | 0.025 (0.038) | 5.04E-01 | -0.056 (0.037) | 1.32E-01 | 0.025 (0.037) | 4.99E-01 | 0.057 (0.054) | 2.91E-01 |
| PCO34:2 | -0.061 (0.055) | 2.68E-01 | **-0.133 (0.065)** | **4.33E-02** | -0.053 (0.162) | 7.42E-01 | -0.028 (0.047) | 5.45E-01 | 0.040 (0.037) | 2.84E-01 | -0.020 (0.037) | 5.95E-01 | 0 (0.036) | 9.98E-01 | -0.033 (0.054) | 5.41E-01 |
| PCO34:3 | -0.027 (0.057) | 6.33E-01 | -0.115 (0.067) | 8.75E-02 | -0.033 (0.160) | 8.35E-01 | 0.020 (0.048) | 6.68E-01 | 0.022 (0.038) | 5.72E-01 | -0.047 (0.037) | 2.07E-01 | 0.049 (0.038) | 1.99E-01 | -0.030 (0.055) | 5.83E-01 |
| PCO36:4 | **-0.081 (0.036)** | **2.44E-02** | **-0.097 (0.044)** | **2.88E-02** | 0.040 (0.105) | 7.04E-01 | -0.016 (0.030) | 5.87E-01 | **0.054 (0.025)** | **2.96E-02** | -0.030 (0.024) | 2.16E-01 | -0.035 (0.025) | 1.53E-01 | -0.031 (0.035) | 3.83E-01 |
| PCO36:5 | **-0.098 (0.044)** | **2.63E-02** | **-0.141 (0.053)** | **8.50E-03** | 0.141 (0.128) | 2.70E-01 | -0.006 (0.036) | 8.59E-01 | **0.071 (0.031)** | **2.16E-02** | -0.036 (0.030) | 2.29E-01 | 0.001 (0.030) | 9.64E-01 | -0.010 (0.044) | 8.17E-01 |
| PCO38:4 | -0.016 (0.046) | 7.35E-01 | **-0.184 (0.057)** | **1.28E-03** | -0.048 (0.134) | 7.17E-01 | 0.017 (0.039) | 6.58E-01 | 0 (0.032) | 9.89E-01 | -0.051 (0.030) | 9.35E-02 | -0.045 (0.031) | 1.50E-01 | -0.079 (0.045) | 7.85E-02 |
| PCO38:5 | -0.060 (0.038) | 1.14E-01 | -0.035 (0.047) | 4.58E-01 | -0.016 (0.110) | 8.82E-01 | 0.022 (0.032) | 4.97E-01 | **0.063 (0.026)** | **1.56E-02** | **-0.057 (0.025)** | **2.44E-02** | -0.008 (0.026) | 7.61E-01 | **-0.075 (0.037)** | **4.12E-02** |
| PCO38:6 | **-0.106 (0.047)** | **2.37E-02** | -0.048 (0.057) | 4.04E-01 | 0.043 (0.137) | 7.53E-01 | **-0.098 (0.039)** | **1.17E-02** | 0.022 (0.032) | 5.03E-01 | -0.042 (0.031) | 1.78E-01 | 0.027 (0.032) | 3.90E-01 | 0.027 (0.047) | 5.57E-01 |
| PE36:2 | -0.033 (0.053) | 5.35E-01 | -0.004 (0.064) | 9.51E-01 | 0.182 (0.145) | 2.09E-01 | 0.058 (0.046) | 2.09E-01 | -0.011 (0.037) | 7.74E-01 | -0.020 (0.035) | 5.71E-01 | 0.010 (0.036) | 7.88E-01 | 0.017 (0.051) | 7.40E-01 |
| PE38:2 | -0.041 (0.041) | 3.23E-01 | -0.048 (0.050) | 3.33E-01 | -0.165 (0.117) | 1.58E-01 | -0.021 (0.034) | 5.32E-01 | -0.029 (0.028) | 2.95E-01 | -0.029 (0.027) | 2.76E-01 | **0.054 (0.027)** | **5.08E-02** | 0.028 (0.040) | 4.82E-01 |
| PE38:4 | **-0.135 (0.062)** | **3.07E-02** | -0.025 (0.075) | 7.42E-01 | 0.249 (0.184) | 1.78E-01 | 0.064 (0.052) | 2.26E-01 | 0.005 (0.043) | 9.16E-01 | 0.011 (0.042) | 7.99E-01 | 0.025 (0.041) | 5.44E-01 | 0.072 (0.059) | 2.24E-01 |
| PEO38:6 | **-0.141 (0.063)** | **2.70E-02** | -0.029 (0.075) | 6.95E-01 | 0.232 (0.203) | 2.54E-01 | 0.012 (0.054) | 8.28E-01 | 0.023 (0.045) | 6.12E-01 | 0.020 (0.043) | 6.44E-01 | 0.064 (0.044) | 1.48E-01 | 0.023 (0.060) | 7.05E-01 |
| SM32:1 | -0.095 (0.052) | 6.77E-02 | -0.117 (0.064) | 6.69E-02 | 0.131 (0.149) | 3.78E-01 | 0.031 (0.044) | 4.79E-01 | 0.011 (0.036) | 7.56E-01 | -0.045 (0.035) | 1.91E-01 | -0.005 (0.035) | 8.97E-01 | -0.011 (0.051) | 8.31E-01 |
| SM34:1 | -0.038 (0.027) | 1.60E-01 | -0.040 (0.033) | 2.35E-01 | -0.002 (0.078) | 9.83E-01 | 0 (0.023) | 9.92E-01 | 0.005 (0.019) | 7.70E-01 | -0.022 (0.018) | 2.25E-01 | 0.018 (0.018) | 3.35E-01 | -0.027 (0.026) | 3.12E-01 |
| SM34:2 | **-0.064 (0.033)** | **5.16E-02** | -0.036 (0.040) | 3.61E-01 | 0.036 (0.095) | 7.06E-01 | -0.030 (0.028) | 2.74E-01 | 0.019 (0.023) | 4.07E-01 | -0.037 (0.021) | 8.23E-02 | 0.015 (0.022) | 5.06E-01 | 0.015 (0.032) | 6.30E-01 |
| SM36:1 | -0.054 (0.041) | 1.81E-01 | -0.064 (0.050) | 2.01E-01 | 0.148 (0.117) | 2.08E-01 | -0.033 (0.034) | 3.39E-01 | 0.010 (0.028) | 7.22E-01 | -0.028 (0.027) | 2.97E-01 | 0.032 (0.027) | 2.38E-01 | -0.026 (0.040) | 5.18E-01 |
| SM36:2 | -0.195 (0.158) | 2.20E-01 | -0.035 (0.199) | 8.61E-01 | 0.596 (0.436) | 1.74E-01 | -0.264 (0.143) | 6.59E-02 | 0.015 (0.114) | 8.97E-01 | -0.149 (0.105) | 1.57E-01 | 0.120 (0.106) | 2.57E-01 | -0.119 (0.155) | 4.41E-01 |
| SM38:1 | -0.031 (0.035) | 3.76E-01 | -0.025 (0.044) | 5.69E-01 | -0.076 (0.103) | 4.62E-01 | 0.002 (0.030) | 9.35E-01 | 0.005 (0.025) | 8.36E-01 | 0.013 (0.024) | 5.84E-01 | 0.041 (0.024) | 8.45E-02 | 0.008 (0.034) | 8.21E-01 |
| SM38:2 | -0.033 (0.061) | 5.96E-01 | 0.034 (0.070) | 6.33E-01 | 0.011 (0.193) | 9.53E-01 | 0.002 (0.053) | 9.71E-01 | 0.018 (0.042) | 6.64E-01 | -0.035 (0.041) | 3.87E-01 | 0.009 (0.041) | 8.30E-01 | -0.03 (0.058) | 6.03E-01 |
| SM40:1 | -0.028 (0.034) | 4.11E-01 | -0.016 (0.042) | 7.14E-01 | -0.008 (0.100) | 9.39E-01 | 0.023 (0.029) | 4.36E-01 | 0.011 (0.024) | 6.29E-01 | -0.004 (0.023) | 8.65E-01 | 0.030 (0.023) | 1.95E-01 | -0.034 (0.033) | 3.05E-01 |
| SM40:2 | **-0.064 (0.033)** | **5.23E-02** | -0.006 (0.040) | 8.80E-01 | -0.080 (0.095) | 4.04E-01 | -0.008 (0.028) | 7.75E-01 | 0.005 (0.023) | 8.10E-01 | -0.026 (0.022) | 2.34E-01 | 0.029 (0.022) | 1.92E-01 | -0.032 (0.032) | 3.17E-01 |
| SM41:1 | -0.061 (0.047) | 1.96E-01 | -0.064 (0.057) | 2.66E-01 | -0.057 (0.134) | 6.69E-01 | -0.008 (0.040) | 8.40E-01 | 0.017 (0.033) | 6.02E-01 | -0.010 (0.032) | 7.46E-01 | 0.016 (0.032) | 6.16E-01 | -0.026 (0.046) | 5.70E-01 |
| SM42:1 | 0.005 (0.050) | 9.17E-01 | -0.081 (0.062) | 1.93E-01 | -0.194 (0.146) | 1.87E-01 | 0.002 (0.042) | 9.64E-01 | 0.039 (0.034) | 2.49E-01 | -0.007 (0.033) | 8.41E-01 | 0.004 (0.033) | 9.15E-01 | -0.017 (0.048) | 7.22E-01 |
| SM42:2 | -0.017 (0.027) | 5.36E-01 | -0.031 (0.033) | 3.47E-01 | -0.066 (0.078) | 3.98E-01 | -0.008 (0.023) | 7.43E-01 | -0.002 (0.019) | 9.06E-01 | 0 (0.018) | 9.98E-01 | 0.029 (0.018) | 1.06E-01 | -0.025 (0.026) | 3.38E-01 |
| SM42:3 | -0.046 (0.027) | 8.33E-02 | -0.020 (0.033) | 5.36E-01 | -0.121 (0.077) | 1.15E-01 | -0.041 (0.023) | 7.19E-02 | 0.005 (0.019) | 7.99E-01 | -0.021 (0.018) | 2.33E-01 | 0.027 (0.018) | 1.38E-01 | -0.014 (0.026) | 5.96E-01 |
| TAG46:1 | 0.001 (0.053) | 9.83E-01 | **-0.153 (0.065)** | **2.04E-02** | **0.690 (0.150)** | **6.00E-06** | 0.084 (0.045) | 6.18E-02 | -0.001 (0.036) | 9.73E-01 | 0.022 (0.036) | 5.46E-01 | 0.021 (0.035) | 5.60E-01 | -0.029 (0.052) | 5.82E-01 |
| TAG46:2 | 0.006 (0.111) | 9.59E-01 | **-0.273 (0.132)** | **3.95E-02** | **0.813 (0.288)** | **5.08E-03** | 0.069 (0.097) | 4.76E-01 | -0.002 (0.077) | 9.78E-01 | 0.053 (0.074) | 4.74E-01 | 0.139 (0.076) | 6.94E-02 | 0.051 (0.108) | 6.39E-01 |
| TAG48:1 | -0.067 (0.092) | 4.64E-01 | -0.153 (0.112) | 1.71E-01 | **0.983 (0.258)** | **1.64E-04** | **0.160 (0.076)** | **3.55E-02** | 0.016 (0.062) | 7.93E-01 | 0.089 (0.061) | 1.45E-01 | -0.013 (0.061) | 8.25E-01 | 0.005 (0.090) | 9.57E-01 |
| TAG48:2 | -0.056 (0.084) | 5.06E-01 | -0.135 (0.102) | 1.85E-01 | **0.763 (0.237)** | **1.39E-03** | **0.160 (0.069)** | **2.09E-02** | 0.029 (0.057) | 6.12E-01 | 0.085 (0.055) | 1.25E-01 | -0.006 (0.055) | 9.07E-01 | 0.006 (0.082) | 9.42E-01 |
| TAG48:3 | -0.108 (0.089) | 2.28E-01 | -0.177 (0.112) | 1.13E-01 | **0.493 (0.251)** | **5.08E-02** | 0.144 (0.077) | 6.17E-02 | -0.015 (0.062) | 8.13E-01 | 0.074 (0.060) | 2.15E-01 | 0.042 (0.060) | 4.89E-01 | 0.017 (0.088) | 8.44E-01 |
| TAG50:1 | -0.085 0.086 | 3.25E-01 | -0.140 (0.105) | 1.84E-01 | **1.003 (0.242)** | **4.10E-05** | 0.092 (0.072) | 1.99E-01 | -0.022 (0.059) | 7.02E-01 | 0.098 (0.057) | 8.62E-02 | 0.011 (0.056) | 8.49E-01 | 0.040 (0.084) | 6.38E-01 |
| TAG50:2 | -0.099 (0.074) | 1.83E-01 | -0.125 (0.090) | 1.69E-01 | **0.691 (0.211)** | **1.17E-03** | **0.123 (0.062)** | **4.64E-02** | 0.004 (0.050) | 9.35E-01 | 0.071 (0.049) | 1.49E-01 | 0.014 (0.049) | 7.80E-01 | 0.029 (0.072) | 6.93E-01 |
| TAG50:3 | -0.092 (0.064) | 1.51E-01 | -0.078 (0.078) | 3.20E-01 | **0.447 (0.183)** | **1.49E-02** | **0.118 (0.053)** | **2.70E-02** | -0.002 (0.043) | 9.54E-01 | 0.049 (0.042) | 2.41E-01 | 0.025 (0.042) | 5.46E-01 | 0.040 (0.062) | 5.17E-01 |
| TAG50:4 | -0.064 (0.069) | 3.57E-01 | -0.104 (0.085) | 2.24E-01 | **0.388 (0.199)** | **5.17E-02** | **0.118 (0.058)** | **4.17E-02** | -0.004 (0.047) | 9.40E-01 | 0.053 (0.045) | 2.43E-01 | 0.012 (0.046) | 7.86E-01 | 0.025 (0.067) | 7.14E-01 |
| TAG51:2 | -0.089 (0.069) | 1.94E-01 | -0.060 (0.084) | 4.75E-01 | **0.457 (0.196)** | **2.01E-02** | **0.117 (0.056)** | **3.56E-02** | -0.017 (0.047) | 7.18E-01 | 0.072 (0.045) | 1.12E-01 | -0.011 (0.045) | 8.16E-01 | 0.042 (0.067) | 5.26E-01 |
| TAG51:3 | **-0.149 (0.065)** | **2.32E-02** | -0.073 (0.081) | 3.70E-01 | 0.272 (0.187) | 1.47E-01 | **0.125 (0.054)** | **2.14E-02** | -0.048 (0.045) | 2.89E-01 | 0.052 (0.044) | 2.37E-01 | 0.033 (0.044) | 4.54E-01 | 0.078 (0.064) | 2.26E-01 |
| TAG52:2 | -0.115 (0.076) | 1.31E-01 | -0.115 (0.092) | 2.14E-01 | **0.643 (0.215)** | **2.99E-03** | 0.102 (0.063) | 1.03E-01 | -0.003 (0.052) | 9.59E-01 | 0.084 (0.050) | 9.38E-02 | 0.023 (0.050) | 6.50E-01 | 0.054 (0.074) | 4.61E-01 |
| TAG52:3 | -0.102 (0.071) | 1.52E-01 | -0.022 (0.087) | 8.03E-01 | 0.337 (0.204) | 1.00E-01 | 0.088 (0.059) | 1.41E-01 | -0.038 (0.048) | 4.35E-01 | 0.036 (0.047) | 4.48E-01 | 0.050 (0.047) | 2.89E-01 | 0.108 (0.069) | 1.17E-01 |
| TAG52:4 | -0.088 (0.065) | 1.74E-01 | 0.001 (0.080) | 9.87E-01 | 0.224 (0.188) | 2.34E-01 | 0.073 (0.055) | 1.85E-01 | -0.039 (0.044) | 3.78E-01 | 0.011 (0.043) | 8.04E-01 | 0.047 (0.043) | 2.72E-01 | 0.099 (0.063) | 1.13E-01 |
| TAG52:5 | -0.088 (0.059) | 1.36E-01 | -0.039 (0.072) | 5.90E-01 | 0.236 (0.171) | 1.67E-01 | 0.073 (0.050) | 1.43E-01 | -0.029 (0.040) | 4.73E-01 | 0.026 (0.039) | 4.99E-01 | 0.046 (0.039) | 2.43E-01 | 0.068 (0.057) | 2.34E-01 |
| TAG52:6 | -0.136 (0.084) | 1.07E-01 | -0.121 (0.105) | 2.50E-01 | 0.325 (0.238) | 1.72E-01 | 0.045 (0.072) | 5.30E-01 | -0.037 (0.058) | 5.30E-01 | 0.069 (0.057) | 2.28E-01 | 0.078 (0.056) | 1.65E-01 | 0.111 (0.083) | 1.79E-01 |
| TAG54:2 | -0.078 (0.054) | 1.50E-01 | -0.086 (0.066) | 1.95E-01 | **0.557 (0.152)** | **2.71E-04** | 0.061 (0.044) | 1.70E-01 | -0.018 (0.037) | 6.33E-01 | **0.078 (0.036)** | **2.97E-02** | 0.035 (0.036) | 3.29E-01 | 0.043 (0.053) | 4.19E-01 |
| TAG54:3 | **-0.110 (0.056)** | **5.24E-02** | -0.062 (0.069) | 3.66E-01 | **0.474 (0.160)** | **3.26E-03** | 0.057 (0.047) | 2.24E-01 | -0.013 (0.038) | 7.29E-01 | 0.065 (0.037) | 8.02E-02 | 0.043 (0.037) | 2.55E-01 | 0.055 (0.055) | 3.20E-01 |
| TAG54:4 | -0.093 (0.059) | 1.14E-01 | -0.025 (0.072) | 7.30E-01 | 0.288 (0.169) | 8.78E-02 | 0.067 (0.049) | 1.72E-01 | -0.031 (0.040) | 4.44E-01 | 0.040 (0.039) | 3.07E-01 | 0.058 (0.039) | 1.39E-01 | 0.079 (0.057) | 1.64E-01 |
| TAG54:5 | -0.082 (0.056) | 1.43E-01 | -0.046 (0.068) | 5.02E-01 | 0.251 (0.162) | 1.22E-01 | 0.066 (0.047) | 1.60E-01 | -0.025 (0.038) | 5.15E-01 | 0.036 (0.037) | 3.29E-01 | 0.048 (0.037) | 1.93E-01 | 0.072 (0.054) | 1.87E-01 |
| TAG54:6 | -0.087 (0.057) | 1.26E-01 | -0.073 (0.069) | 2.94E-01 | 0.291 (0.163) | 7.50E-02 | 0.041 (0.047) | 3.85E-01 | -0.029 (0.039) | 4.50E-01 | 0.019 (0.037) | 6.05E-01 | 0.063 (0.037) | 8.76E-02 | 0.084 (0.055) | 1.25E-01 |
| TAG54:7 | -0.112 (0.071) | 1.14E-01 | -0.100 (0.087) | 2.50E-01 | 0.303 (0.204) | 1.39E-01 | -0.006 (0.060) | 9.14E-01 | -0.006 (0.048) | 9.02E-01 | 0.067 (0.047) | 1.53E-01 | 0.075 (0.047) | 1.13E-01 | 0.104 (0.069) | 1.32E-01 |
| TAG56:5 | **-0.119 (0.054)** | **2.86E-02** | -0.101 (0.066) | 1.28E-01 | 0.301 (0.156) | 5.52E-02 | 0.072 (0.044) | 1.04E-01 | 0.008 (0.037) | 8.35E-01 | 0.065 (0.036) | 6.89E-02 | 0.068 (0.036) | 5.84E-02 | 0.081 (0.053) | 1.22E-01 |
| TAG56:6 | **-0.106 (0.049)** | **3.22E-02** | -0.076 (0.060) | 2.04E-01 | **0.297 (0.142)** | **3.62E-02** | 0.059 (0.041) | 1.53E-01 | 0.001 (0.034) | 9.79E-01 | 0.020 (0.033) | 5.48E-01 | 0.042 (0.033) | 2.00E-01 | 0.087 (0.048) | 6.90E-02 |
| TAG56:7 | -0.113 (0.059) | 5.72E-02 | -0.069 (0.072) | 3.42E-01 | **0.338 (0.170)** | **4.77E-02** | -0.026 (0.049) | 5.96E-01 | -0.006 (0.040) | 8.75E-01 | 0.018 (0.039) | 6.37E-01 | **0.094 (0.039)** | **1.65E-02** | **0.117 (0.057)** | **4.16E-02** |
| TAG56:8 | -0.106 (0.071) | 1.35E-01 | -0.028 (0.087) | 7.53E-01 | 0.218 (0.206) | 2.91E-01 | -0.025 (0.060) | 6.77E-01 | -0.005 (0.048) | 9.16E-01 | 0.015 (0.047) | 7.49E-01 | **0.112 (0.047)** | **1.81E-02** | **0.147 (0.069)** | **3.32E-02** |
| TAG58:7 | -0.152 (0.088) | 8.42E-02 | -0.088 (0.100) | 3.78E-01 | 0.358 (0.209) | 8.81E-02 | 0.019 (0.072) | 7.91E-01 | -0.066 (0.057) | 2.48E-01 | 0.008 (0.056) | 8.80E-01 | 0.030 (0.057) | 6.01E-01 | 0.121 (0.079) | 1.25E-01 |
| TAG58:8 | -0.125 (0.073) | 8.76E-02 | -0.065 (0.089) | 4.67E-01 | 0.267 (0.207) | 1.98E-01 | -0.025 (0.062) | 6.81E-01 | -0.012 (0.050) | 8.13E-01 | 0.045 (0.049) | 3.59E-01 | **0.141 (0.049)** | **4.30E-03** | **0.172 (0.071)** | **1.59E-02** |
| TAG58:9 | -0.051 (0.087) | 5.57E-01 | 0.039 (0.101) | 6.98E-01 | -0.101 (0.227) | 6.57E-01 | -0.117 (0.073) | 1.08E-01 | 0.023 (0.057) | 6.91E-01 | 0.021 (0.057) | 7.15E-01 | **0.148 (0.057)** | **9.42E-03** | **0.190 (0.081)** | **1.87E-02** |
| TAG58:10 | **-0.191 (0.092)** | **3.85E-02** | 0.029 (0.110) | 7.91E-01 | -0.069 (0.257) | 7.88E-01 | -0.092 (0.077) | 2.31E-01 | 0.058 (0.064) | 3.66E-01 | -0.041 (0.061) | 5.01E-01 | 0.118 (0.062) | 5.73E-02 | 0.071 (0.086) | 4.08E-01 |

Linear regressions were performed between the CAD-associated locus (with the CAD-associated allele coded) and the lipid species after log transformation adjusting for age and sex. β < 0: the CAD risk allele negatively correlates with the associated lipid specie. β > 0: the CAD risk allele positively correlates with the associated lipid specie. Chol, cholesterylester; DAG, diacylglyceride; LPC, lysophosphatidylcholine; PC, phosphatidyl-choline; PC-O, phosphatidylcholine ether; PE, phosphatidylethanolamine; PE-O, phosphatidylethanolamine ether; SM, sphingomyelin; TAG, triacylglyceride.
