# Supplementary material for: Plasma Lipid Composition and Risk of Developing Cardiovascular Disease
Source: PLoS One. 2013 Aug 15;8(8):e71846. doi: 10.1371/journal.pone.0071846 (PMC3744469; doi:10.1371/journal.pone.0071846)
Supplement: Table S8 — Estimated q-values of the tests performed to study the association between CAD-associated gene variants and the lipid species. (DOCX) [file pone.0071846.s011.docx]

**Supplementary Table S8.** Estimated q-values of the tests performed to study the association between CAD-associated gene variants and the lipid species.

|  | rs6725887_WDR12 | | rs17114036_PPAP2B | | rs3798220_ LPA | | rs646776_ SORT1 | | rs11556924_ZC3HC1 | | rs12936587_PEMT, RASD1, SMCR3 | | rs9349379_PHACTR1 | | rs964184_ ZNF259,APOA5-A4-C3-A1 | |
| --- | --- | --- | --- | --- | --- | --- | --- | --- | --- | --- | --- | --- | --- | --- | --- | --- |
| Lipid specie | *P* | q-value | *P* | q-value | *P* | q-value | *P* | q-value | *P* | q-value | *P* | q-value | *P* | q-value | *P* | q-value |
| Chol16:1 | 2.47E-01 | 8.19E-01 | **1.72E-02** | 7.42E-01 | 8.19E-02 | 7.77E-01 | 1.91E-01 | 7.98E-01 | 1.10E-01 | 7.88E-01 | 3.04E-01 | 8.31E-01 | 6.62E-01 | 8.86E-01 | 1.60E-01 | 7.98E-01 |
| Chol16:0 | **4.50E-02** | 7.52E-01 | 2.38E-01 | 8.13E-01 | 2.63E-01 | 8.24E-01 | 7.17E-01 | 8.87E-01 | 3.12E-01 | 8.31E-01 | 5.52E-01 | 8.86E-01 | 2.29E-01 | 8.12E-01 | 6.38E-01 | 8.86E-01 |
| Chol18:1 | 6.56E-02 | 7.52E-01 | **2.50E-02** | 7.42E-01 | 4.40E-01 | 8.79E-01 | 8.58E-01 | 9.15E-01 | **5.04E-02** | 7.52E-01 | 4.33E-01 | 8.76E-01 | 8.76E-01 | 9.15E-01 | 1.36E-01 | 7.98E-01 |
| Chol18:2 | 4.79E-01 | 8.79E-01 | 2.04E-01 | 8.01E-01 | 6.94E-02 | 7.52E-01 | 9.67E-01 | 9.16E-01 | 9.23E-01 | 9.15E-01 | 3.65E-01 | 8.51E-01 | 3.23E-01 | 8.40E-01 | 3.27E-01 | 8.42E-01 |
| Chol18:3 | **3.28E-02** | 7.52E-01 | 3.21E-01 | 8.39E-01 | 9.26E-01 | 9.15E-01 | 6.01E-01 | 8.86E-01 | 7.48E-02 | 7.71E-01 | 5.42E-01 | 8.86E-01 | 3.27E-01 | 8.42E-01 | 4.72E-01 | 8.79E-01 |
| Chol20:3 | 4.36E-01 | 8.76E-01 | **1.08E-02** | 7.42E-01 | 8.00E-01 | 9.04E-01 | 3.62E-01 | 8.51E-01 | 2.33E-01 | 8.12E-01 | 5.18E-01 | 8.86E-01 | 3.56E-01 | 8.51E-01 | 6.51E-01 | 8.86E-01 |
| Chol20:4 | 9.97E-02 | 7.88E-01 | 4.18E-01 | 8.67E-01 | 8.82E-01 | 9.15E-01 | 5.03E-01 | 8.86E-01 | 5.44E-02 | 7.52E-01 | 9.48E-01 | 9.15E-01 | 8.50E-01 | 9.13E-01 | 8.22E-01 | 9.08E-01 |
| Chol20:5 | 2.53E-01 | 8.20E-01 | 9.03E-01 | 9.15E-01 | 5.72E-01 | 8.86E-01 | 9.82E-02 | 7.88E-01 | 2.62E-01 | 8.24E-01 | 4.08E-01 | 8.60E-01 | 6.26E-01 | 8.86E-01 | 2.07E-01 | 8.03E-01 |
| Chol22:6 | 2.98E-01 | 8.31E-01 | 7.00E-01 | 8.87E-01 | 4.80E-01 | 8.79E-01 | 1.11E-01 | 7.88E-01 | 2.83E-01 | 8.28E-01 | 6.51E-01 | 8.86E-01 | 6.94E-02 | 7.52E-01 | 1.96E-01 | 7.98E-01 |
| DAG36:2 | 1.97E-01 | 7.98E-01 | 5.14E-01 | 8.86E-01 | 2.37E-01 | 8.13E-01 | 2.69E-01 | 8.24E-01 | 2.52E-01 | 8.19E-01 | 2.32E-01 | 8.12E-01 | 1.80E-01 | 7.98E-01 | 2.18E-01 | 8.12E-01 |
| LPC16:0 | 1.17E-01 | 7.94E-01 | **5.56E-02** | 7.52E-01 | 1.67E-01 | 7.98E-01 | 5.17E-01 | 8.86E-01 | 5.94E-01 | 8.86E-01 | **3.09E-02** | 7.42E-01 | 7.37E-01 | 8.88E-01 | 2.70E-01 | 8.24E-01 |
| LPC18:1 | 5.90E-02 | 7.52E-01 | **3.10E-02** | 7.42E-01 | 5.15E-01 | 8.86E-01 | 1.13E-01 | 7.88E-01 | 7.33E-01 | 8.87E-01 | 2.06E-01 | 8.03E-01 | 7.76E-01 | 9.03E-01 | 1.49E-01 | 7.98E-01 |
| LPC18:3 | **1.76E-02** | 7.42E-01 | 4.79E-01 | 8.79E-01 | 8.60E-01 | 9.15E-01 | 9.23E-02 | 7.83E-01 | 3.74E-01 | 8.55E-01 | 1.04E-01 | 7.88E-01 | 7.11E-01 | 8.87E-01 | 2.35E-01 | 8.12E-01 |
| LPC18:0 | **4.22E-02** | 7.52E-01 | 4.32E-01 | 8.76E-01 | 5.06E-01 | 8.86E-01 | 3.44E-01 | 8.48E-01 | 1.58E-01 | 7.98E-01 | 5.63E-02 | 7.52E-01 | 7.32E-01 | 8.87E-01 | 9.53E-01 | 9.15E-01 |
| LPC20:4 | 8.95E-01 | 9.15E-01 | 3.64E-01 | 8.51E-01 | 8.42E-01 | 9.13E-01 | **1.16E-02** | 7.42E-01 | 2.87E-01 | 8.30E-01 | **4.65E-02** | 7.52E-01 | 4.65E-01 | 8.79E-01 | 7.77E-01 | 9.03E-01 |
| PC32:1 | 2.78E-01 | 8.26E-01 | **1.41E-02** | 7.42E-01 | 5.36E-02 | 7.52E-01 | 8.80E-02 | 7.77E-01 | **2.63E-02** | 7.42E-01 | 2.89E-01 | 8.30E-01 | 2.94E-01 | 8.30E-01 | 1.37E-01 | 7.98E-01 |
| PC32:0 | 6.75E-02 | 7.52E-01 | **5.30E-02** | 7.52E-01 | 5.87E-02 | 7.52E-01 | 8.28E-01 | 9.10E-01 | 1.72E-01 | 7.98E-01 | 5.49E-01 | 8.86E-01 | 9.86E-01 | 9.19E-01 | 5.98E-01 | 8.86E-01 |
| PC34:1 | 6.43E-02 | 7.52E-01 | **7.04E-03** | 7.42E-01 | **1.08E-02** | 7.42E-01 | 7.07E-01 | 8.87E-01 | 6.19E-02 | 7.52E-01 | 7.66E-01 | 9.02E-01 | 6.49E-01 | 8.86E-01 | 1.33E-01 | 7.98E-01 |
| PC34:2 | 7.60E-01 | 8.99E-01 | 4.01E-01 | 8.60E-01 | 2.98E-01 | 8.31E-01 | 4.56E-01 | 8.79E-01 | 6.66E-01 | 8.86E-01 | 2.85E-01 | 8.28E-01 | 3.90E-01 | 8.59E-01 | 6.89E-01 | 8.86E-01 |
| PC34:3 | **2.33E-02** | 7.42E-01 | 1.46E-01 | 7.98E-01 | 3.01E-01 | 8.31E-01 | 5.78E-01 | 8.86E-01 | 7.99E-02 | 7.77E-01 | 6.66E-01 | 8.86E-01 | 7.37E-01 | 8.88E-01 | 6.51E-02 | 7.52E-01 |
| PC36:2 | 5.19E-01 | 8.86E-01 | 2.28E-01 | 8.12E-01 | 7.80E-02 | 7.77E-01 | 6.25E-01 | 8.86E-01 | 9.17E-01 | 9.15E-01 | 2.70E-01 | 8.24E-01 | 5.94E-01 | 8.86E-01 | 8.48E-01 | 9.13E-01 |
| PC36:3 | **2.82E-02** | 7.42E-01 | **1.25E-02** | 7.42E-01 | 1.18E-01 | 7.94E-01 | 6.36E-01 | 8.86E-01 | 1.10E-01 | 7.88E-01 | 2.53E-01 | 8.20E-01 | 9.89E-01 | 9.19E-01 | 3.83E-01 | 8.58E-01 |
| PC36:4 | **2.86E-02** | 7.42E-01 | **1.20E-02** | 7.42E-01 | 2.22E-01 | 8.12E-01 | 4.51E-01 | 8.79E-01 | **4.51E-02** | 7.52E-01 | 5.33E-01 | 8.86E-01 | 7.50E-01 | 8.95E-01 | 9.45E-01 | 9.15E-01 |
| PC36:5 | 9.75E-02 | 7.88E-01 | 2.04E-01 | 8.01E-01 | 4.48E-01 | 8.79E-01 | 3.14E-01 | 8.32E-01 | 2.63E-01 | 8.24E-01 | **3.74E-02** | 7.52E-01 | 4.31E-01 | 8.76E-01 | 9.07E-01 | 9.15E-01 |
| PC38:3 | 1.75E-01 | 7.98E-01 | **3.34E-03** | 6.08E-01 | 6.27E-02 | 7.52E-01 | 1.87E-01 | 7.98E-01 | 8.15E-01 | 9.07E-01 | 4.24E-01 | 8.73E-01 | 2.44E-01 | 8.19E-01 | 8.02E-01 | 9.05E-01 |
| PC38:4 | **1.88E-02** | 7.42E-01 | **4.65E-03** | 6.51E-01 | 1.16E-01 | 7.90E-01 | 6.84E-01 | 8.86E-01 | 1.64E-01 | 7.98E-01 | 2.65E-01 | 8.24E-01 | 5.69E-01 | 8.86E-01 | 9.03E-01 | 9.15E-01 |
| PC38:5 | **7.01E-03** | 7.42E-01 | **8.81E-03** | 7.42E-01 | 1.21E-01 | 7.98E-01 | 5.27E-01 | 8.86E-01 | 1.42E-01 | 7.98E-01 | **4.32E-02** | 7.52E-01 | 6.14E-01 | 8.86E-01 | 6.74E-01 | 8.86E-01 |
| PC38:6 | 7.58E-02 | 7.71E-01 | **3.58E-02** | 7.52E-01 | 4.35E-01 | 8.76E-01 | 8.17E-02 | 7.77E-01 | 3.21E-01 | 8.39E-01 | 1.41E-01 | 7.98E-01 | 9.70E-02 | 7.88E-01 | 1.85E-01 | 7.98E-01 |
| PC38:7 | 3.72E-01 | 8.54E-01 | 4.06E-01 | 8.60E-01 | 9.04E-01 | 9.15E-01 | 4.80E-01 | 8.79E-01 | **4.94E-02** | 7.52E-01 | 9.41E-01 | 9.15E-01 | 8.83E-01 | 9.15E-01 | 5.83E-01 | 8.86E-01 |
| PC40:6 | 9.42E-02 | 7.83E-01 | **2.32E-02** | 7.42E-01 | 9.89E-02 | 7.88E-01 | 1.37E-01 | 7.98E-01 | 7.41E-01 | 8.90E-01 | 3.52E-01 | 8.49E-01 | 2.88E-01 | 8.30E-01 | 4.38E-01 | 8.79E-01 |
| PC40:7 | **7.16E-03** | 7.42E-01 | 1.59E-01 | 7.98E-01 | 9.18E-01 | 9.15E-01 | 3.44E-01 | 8.48E-01 | 4.65E-01 | 8.79E-01 | 2.95E-01 | 8.30E-01 | 7.48E-01 | 8.95E-01 | 4.81E-01 | 8.79E-01 |
| PC40:8 | **2.47E-02** | 7.42E-01 | 6.08E-02 | 7.52E-01 | 8.66E-01 | 9.15E-01 | **4.20E-02** | 7.52E-01 | 5.04E-01 | 8.86E-01 | 1.32E-01 | 7.98E-01 | 4.99E-01 | 8.86E-01 | 2.91E-01 | 8.30E-01 |
| PCO34:2 | 2.68E-01 | 8.24E-01 | **4.33E-02** | 7.52E-01 | 7.42E-01 | 8.90E-01 | 5.45E-01 | 8.86E-01 | 2.84E-01 | 8.28E-01 | 5.95E-01 | 8.86E-01 | 9.98E-01 | 9.19E-01 | 5.41E-01 | 8.86E-01 |
| PCO34:3 | 6.33E-01 | 8.86E-01 | 8.75E-02 | 7.77E-01 | 8.35E-01 | 9.10E-01 | 6.68E-01 | 8.86E-01 | 5.72E-01 | 8.86E-01 | 2.07E-01 | 8.03E-01 | 1.99E-01 | 7.98E-01 | 5.83E-01 | 8.86E-01 |
| PCO36:4 | **2.44E-02** | 7.42E-01 | **2.88E-02** | 7.42E-01 | 7.04E-01 | 8.87E-01 | 5.87E-01 | 8.86E-01 | **2.96E-02** | 7.42E-01 | 2.16E-01 | 8.12E-01 | 1.53E-01 | 7.98E-01 | 3.83E-01 | 8.58E-01 |
| PCO36:5 | **2.63E-02** | 7.42E-01 | **8.50E-03** | 7.42E-01 | 2.70E-01 | 8.24E-01 | 8.59E-01 | 9.15E-01 | **2.16E-02** | 7.42E-01 | 2.29E-01 | 8.12E-01 | 9.64E-01 | 9.15E-01 | 8.17E-01 | 9.07E-01 |
| PCO38:4 | 7.35E-01 | 8.88E-01 | **1.28E-03** | 3.61E-01 | 7.17E-01 | 8.87E-01 | 6.58E-01 | 8.86E-01 | 9.89E-01 | 9.19E-01 | 9.35E-02 | 7.83E-01 | 1.50E-01 | 7.98E-01 | 7.85E-02 | 7.77E-01 |
| PCO38:5 | 1.14E-01 | 7.88E-01 | 4.58E-01 | 8.79E-01 | 8.82E-01 | 9.15E-01 | 4.97E-01 | 8.86E-01 | **1.56E-02** | 7.42E-01 | **2.44E-02** | 7.42E-01 | 7.61E-01 | 8.99E-01 | **4.12E-02** | 7.52E-01 |
| PCO38:6 | **2.37E-02** | 7.42E-01 | 4.04E-01 | 8.60E-01 | 7.53E-01 | 8.95E-01 | **1.17E-02** | 7.42E-01 | 5.03E-01 | 8.86E-01 | 1.78E-01 | 7.98E-01 | 3.90E-01 | 8.59E-01 | 5.57E-01 | 8.86E-01 |
| PE36:2 | 5.35E-01 | 8.86E-01 | 9.51E-01 | 9.15E-01 | 2.09E-01 | 8.03E-01 | 2.09E-01 | 8.03E-01 | 7.74E-01 | 9.03E-01 | 5.71E-01 | 8.86E-01 | 7.88E-01 | 9.03E-01 | 7.40E-01 | 8.90E-01 |
| PE38:2 | 3.23E-01 | 8.40E-01 | 3.33E-01 | 8.43E-01 | 1.58E-01 | 7.98E-01 | 5.32E-01 | 8.86E-01 | 2.95E-01 | 8.30E-01 | 2.76E-01 | 8.26E-01 | **5.08E-02** | 7.52E-01 | 4.82E-01 | 8.79E-01 |
| PE38:4 | **3.07E-02** | 7.42E-01 | 7.42E-01 | 8.90E-01 | 1.78E-01 | 7.98E-01 | 2.26E-01 | 8.12E-01 | 9.16E-01 | 9.15E-01 | 7.99E-01 | 9.04E-01 | 5.44E-01 | 8.86E-01 | 2.24E-01 | 8.12E-01 |
| PEO38:6 | **2.70E-02** | 7.42E-01 | 6.95E-01 | 8.87E-01 | 2.54E-01 | 8.21E-01 | 8.28E-01 | 9.10E-01 | 6.12E-01 | 8.86E-01 | 6.44E-01 | 8.86E-01 | 1.48E-01 | 7.98E-01 | 7.05E-01 | 8.87E-01 |
| SM32:1 | 6.77E-02 | 7.52E-01 | 6.69E-02 | 7.52E-01 | 3.78E-01 | 8.56E-01 | 4.79E-01 | 8.79E-01 | 7.56E-01 | 8.96E-01 | 1.91E-01 | 7.98E-01 | 8.97E-01 | 9.15E-01 | 8.31E-01 | 9.10E-01 |
| SM34:1 | 1.60E-01 | 7.98E-01 | 2.35E-01 | 8.12E-01 | 9.83E-01 | 9.19E-01 | 9.92E-01 | 9.19E-01 | 7.70E-01 | 9.03E-01 | 2.25E-01 | 8.12E-01 | 3.35E-01 | 8.43E-01 | 3.12E-01 | 8.31E-01 |
| SM34:2 | **5.16E-02** | 7.52E-01 | 3.61E-01 | 8.51E-01 | 7.06E-01 | 8.87E-01 | 2.74E-01 | 8.26E-01 | 4.07E-01 | 8.60E-01 | 8.23E-02 | 7.77E-01 | 5.06E-01 | 8.86E-01 | 6.30E-01 | 8.86E-01 |
| SM36:1 | 1.81E-01 | 7.98E-01 | 2.01E-01 | 7.98E-01 | 2.08E-01 | 8.03E-01 | 3.39E-01 | 8.43E-01 | 7.22E-01 | 8.87E-01 | 2.97E-01 | 8.31E-01 | 2.38E-01 | 8.13E-01 | 5.18E-01 | 8.86E-01 |
| SM36:2 | 2.20E-01 | 8.12E-01 | 8.61E-01 | 9.15E-01 | 1.74E-01 | 7.98E-01 | 6.59E-02 | 7.52E-01 | 8.97E-01 | 9.15E-01 | 1.57E-01 | 7.98E-01 | 2.57E-01 | 8.23E-01 | 4.41E-01 | 8.79E-01 |
| SM38:1 | 3.76E-01 | 8.56E-01 | 5.69E-01 | 8.86E-01 | 4.62E-01 | 8.79E-01 | 9.35E-01 | 9.15E-01 | 8.36E-01 | 9.10E-01 | 5.84E-01 | 8.86E-01 | 8.45E-02 | 7.77E-01 | 8.21E-01 | 9.08E-01 |
| SM38:2 | 5.96E-01 | 8.86E-01 | 6.33E-01 | 8.86E-01 | 9.53E-01 | 9.15E-01 | 9.71E-01 | 9.18E-01 | 6.64E-01 | 8.86E-01 | 3.87E-01 | 8.59E-01 | 8.30E-01 | 9.10E-01 | 6.03E-01 | 8.86E-01 |
| SM40:1 | 4.11E-01 | 8.60E-01 | 7.14E-01 | 8.87E-01 | 9.39E-01 | 9.15E-01 | 4.36E-01 | 8.76E-01 | 6.29E-01 | 8.86E-01 | 8.65E-01 | 9.15E-01 | 1.95E-01 | 7.98E-01 | 3.05E-01 | 8.31E-01 |
| SM40:2 | **5.23E-02** | 7.52E-01 | 8.80E-01 | 9.15E-01 | 4.04E-01 | 8.60E-01 | 7.75E-01 | 9.03E-01 | 8.10E-01 | 9.06E-01 | 2.34E-01 | 8.12E-01 | 1.92E-01 | 7.98E-01 | 3.17E-01 | 8.38E-01 |
| SM41:1 | 1.96E-01 | 7.98E-01 | 2.66E-01 | 8.24E-01 | 6.69E-01 | 8.86E-01 | 8.40E-01 | 9.12E-01 | 6.02E-01 | 8.86E-01 | 7.46E-01 | 8.93E-01 | 6.16E-01 | 8.86E-01 | 5.70E-01 | 8.86E-01 |
| SM42:1 | 9.17E-01 | 9.15E-01 | 1.93E-01 | 7.98E-01 | 1.87E-01 | 7.98E-01 | 9.64E-01 | 9.15E-01 | 2.49E-01 | 8.19E-01 | 8.41E-01 | 9.12E-01 | 9.15E-01 | 9.15E-01 | 7.22E-01 | 8.87E-01 |
| SM42:2 | 5.36E-01 | 8.86E-01 | 3.47E-01 | 8.49E-01 | 3.98E-01 | 8.60E-01 | 7.43E-01 | 8.90E-01 | 9.06E-01 | 9.15E-01 | 9.98E-01 | 9.19E-01 | 1.06E-01 | 7.88E-01 | 3.38E-01 | 8.43E-01 |
| SM42:3 | 8.33E-02 | 7.77E-01 | 5.36E-01 | 8.86E-01 | 1.15E-01 | 7.88E-01 | 7.19E-02 | 7.70E-01 | 7.99E-01 | 9.04E-01 | 2.33E-01 | 8.12E-01 | 1.38E-01 | 7.98E-01 | 5.96E-01 | 8.86E-01 |
| TAG46:1 | 9.83E-01 | 9.19E-01 | **2.04E-02** | 7.42E-01 | **6.00E-06** | **1.09E-02** | 6.18E-02 | 7.52E-01 | 9.73E-01 | 9.18E-01 | 5.46E-01 | 8.86E-01 | 5.60E-01 | 8.86E-01 | 5.82E-01 | 8.86E-01 |
| TAG46:2 | 9.59E-01 | 9.15E-01 | **3.95E-02** | 7.52E-01 | **5.08E-03** | 6.60E-01 | 4.76E-01 | 8.79E-01 | 9.78E-01 | 9.19E-01 | 4.74E-01 | 8.79E-01 | 6.94E-02 | 7.52E-01 | 6.39E-01 | 8.86E-01 |
| TAG48:1 | 4.64E-01 | 8.79E-01 | 1.71E-01 | 7.98E-01 | **1.64E-04** | **9.95E-02** | **3.55E-02** | 7.52E-01 | 7.93E-01 | 9.04E-01 | 1.45E-01 | 7.98E-01 | 8.25E-01 | 9.09E-01 | 9.57E-01 | 9.15E-01 |
| TAG48:2 | 5.06E-01 | 8.86E-01 | 1.85E-01 | 7.98E-01 | **1.39E-03** | 3.61E-01 | **2.09E-02** | 7.42E-01 | 6.12E-01 | 8.86E-01 | 1.25E-01 | 7.98E-01 | 9.07E-01 | 9.15E-01 | 9.42E-01 | 9.15E-01 |
| TAG48:3 | 2.28E-01 | 8.12E-01 | 1.13E-01 | 7.88E-01 | **5.08E-02** | 7.52E-01 | 6.17E-02 | 7.52E-01 | 8.13E-01 | 9.07E-01 | 2.15E-01 | 8.12E-01 | 4.89E-01 | 8.80E-01 | 8.44E-01 | 9.13E-01 |
| TAG50:1 | 3.25E-01 | 8.42E-01 | 1.84E-01 | 7.98E-01 | **4.10E-05** | **3.73E-02** | 1.99E-01 | 7.98E-01 | 7.02E-01 | 8.87E-01 | 8.62E-02 | 7.77E-01 | 8.49E-01 | 9.13E-01 | 6.38E-01 | 8.86E-01 |
| TAG50:2 | 1.83E-01 | 7.98E-01 | 1.69E-01 | 7.98E-01 | **1.17E-03** | 3.61E-01 | **4.64E-02** | 7.52E-01 | 9.35E-01 | 9.15E-01 | 1.49E-01 | 7.98E-01 | 7.80E-01 | 9.03E-01 | 6.93E-01 | 8.87E-01 |
| TAG50:3 | 1.51E-01 | 7.98E-01 | 3.20E-01 | 8.39E-01 | **1.49E-02** | 7.42E-01 | **2.70E-02** | 7.42E-01 | 9.54E-01 | 9.15E-01 | 2.41E-01 | 8.19E-01 | 5.46E-01 | 8.86E-01 | 5.17E-01 | 8.86E-01 |
| TAG50:4 | 3.57E-01 | 8.51E-01 | 2.24E-01 | 8.12E-01 | **5.17E-02** | 7.52E-01 | **4.17E-02** | 7.52E-01 | 9.40E-01 | 9.15E-01 | 2.43E-01 | 8.19E-01 | 7.86E-01 | 9.03E-01 | 7.14E-01 | 8.87E-01 |
| TAG51:2 | 1.94E-01 | 7.98E-01 | 4.75E-01 | 8.79E-01 | **2.01E-02** | 7.42E-01 | **3.56E-02** | 7.52E-01 | 7.18E-01 | 8.87E-01 | 1.12E-01 | 7.88E-01 | 8.16E-01 | 9.07E-01 | 5.26E-01 | 8.86E-01 |
| TAG51:3 | **2.32E-02** | 7.42E-01 | 3.70E-01 | 8.54E-01 | 1.47E-01 | 7.98E-01 | **2.14E-02** | 7.42E-01 | 2.89E-01 | 8.30E-01 | 2.37E-01 | 8.13E-01 | 4.54E-01 | 8.79E-01 | 2.26E-01 | 8.12E-01 |
| TAG52:2 | 1.31E-01 | 7.98E-01 | 2.14E-01 | 8.12E-01 | **2.99E-03** | 6.08E-01 | 1.03E-01 | 7.88E-01 | 9.59E-01 | 9.15E-01 | 9.38E-02 | 7.83E-01 | 6.50E-01 | 8.86E-01 | 4.61E-01 | 8.79E-01 |
| TAG52:3 | 1.52E-01 | 7.98E-01 | 8.03E-01 | 9.06E-01 | 1.00E-01 | 7.88E-01 | 1.41E-01 | 7.98E-01 | 4.35E-01 | 8.76E-01 | 4.48E-01 | 8.79E-01 | 2.89E-01 | 8.30E-01 | 1.17E-01 | 7.94E-01 |
| TAG52:4 | 1.74E-01 | 7.98E-01 | 9.87E-01 | 9.19E-01 | 2.34E-01 | 8.12E-01 | 1.85E-01 | 7.98E-01 | 3.78E-01 | 8.56E-01 | 8.04E-01 | 9.06E-01 | 2.72E-01 | 8.26E-01 | 1.13E-01 | 7.88E-01 |
| TAG52:5 | 1.36E-01 | 7.98E-01 | 5.90E-01 | 8.86E-01 | 1.67E-01 | 7.98E-01 | 1.43E-01 | 7.98E-01 | 4.73E-01 | 8.79E-01 | 4.99E-01 | 8.86E-01 | 2.43E-01 | 8.19E-01 | 2.34E-01 | 8.12E-01 |
| TAG52:6 | 1.07E-01 | 7.88E-01 | 2.50E-01 | 8.19E-01 | 1.72E-01 | 7.98E-01 | 5.30E-01 | 8.86E-01 | 5.30E-01 | 8.86E-01 | 2.28E-01 | 8.12E-01 | 1.65E-01 | 7.98E-01 | 1.79E-01 | 7.98E-01 |
| TAG54:2 | 1.50E-01 | 7.98E-01 | 1.95E-01 | 7.98E-01 | **2.71E-04** | 1.23E-01 | 1.70E-01 | 7.98E-01 | 6.33E-01 | 8.86E-01 | **2.97E-02** | 7.42E-01 | 3.29E-01 | 8.42E-01 | 4.19E-01 | 8.67E-01 |
| TAG54:3 | **5.24E-02** | 7.52E-01 | 3.66E-01 | 8.51E-01 | **3.26E-03** | 6.08E-01 | 2.24E-01 | 8.12E-01 | 7.29E-01 | 8.87E-01 | 8.02E-02 | 7.77E-01 | 2.55E-01 | 8.23E-01 | 3.20E-01 | 8.39E-01 |
| TAG54:4 | 1.14E-01 | 7.88E-01 | 7.30E-01 | 8.87E-01 | 8.78E-02 | 7.77E-01 | 1.72E-01 | 7.98E-01 | 4.44E-01 | 8.79E-01 | 3.07E-01 | 8.31E-01 | 1.39E-01 | 7.98E-01 | 1.64E-01 | 7.98E-01 |
| TAG54:5 | 1.43E-01 | 7.98E-01 | 5.02E-01 | 8.86E-01 | 1.22E-01 | 7.98E-01 | 1.60E-01 | 7.98E-01 | 5.15E-01 | 8.86E-01 | 3.29E-01 | 8.42E-01 | 1.93E-01 | 7.98E-01 | 1.87E-01 | 7.98E-01 |
| TAG54:6 | 1.26E-01 | 7.98E-01 | 2.94E-01 | 8.30E-01 | 7.50E-02 | 7.71E-01 | 3.85E-01 | 8.58E-01 | 4.50E-01 | 8.79E-01 | 6.05E-01 | 8.86E-01 | 8.76E-02 | 7.77E-01 | 1.25E-01 | 7.98E-01 |
| TAG54:7 | 1.14E-01 | 7.88E-01 | 2.50E-01 | 8.19E-01 | 1.39E-01 | 7.98E-01 | 9.14E-01 | 9.15E-01 | 9.02E-01 | 9.15E-01 | 1.53E-01 | 7.98E-01 | 1.13E-01 | 7.88E-01 | 1.32E-01 | 7.98E-01 |
| TAG56:5 | **2.86E-02** | 7.42E-01 | 1.28E-01 | 7.98E-01 | 5.52E-02 | 7.52E-01 | 1.04E-01 | 7.88E-01 | 8.35E-01 | 9.10E-01 | 6.89E-02 | 7.52E-01 | 5.84E-02 | 7.52E-01 | 1.22E-01 | 7.98E-01 |
| TAG56:6 | **3.22E-02** | 7.50E-01 | 2.04E-01 | 8.01E-01 | **3.62E-02** | 7.52E-01 | 1.53E-01 | 7.98E-01 | 9.79E-01 | 9.19E-01 | 5.48E-01 | 8.86E-01 | 2.00E-01 | 7.98E-01 | 6.90E-02 | 7.52E-01 |
| TAG56:7 | 5.72E-02 | 7.52E-01 | 3.42E-01 | 8.47E-01 | **4.77E-02** | 7.52E-01 | 5.96E-01 | 8.86E-01 | 8.75E-01 | 9.15E-01 | 6.37E-01 | 8.86E-01 | **1.65E-02** | 7.42E-01 | **4.16E-02** | 7.52E-01 |
| TAG56:8 | 1.35E-01 | 7.98E-01 | 7.53E-01 | 8.95E-01 | 2.91E-01 | 8.30E-01 | 6.77E-01 | 8.86E-01 | 9.16E-01 | 9.15E-01 | 7.49E-01 | 8.95E-01 | **1.81E-02** | 7.42E-01 | **3.32E-02** | 7.52E-01 |
| TAG58:7 | 8.42E-02 | 7.77E-01 | 3.78E-01 | 8.56E-01 | 8.81E-02 | 7.77E-01 | 7.91E-01 | 9.04E-01 | 2.48E-01 | 8.19E-01 | 8.80E-01 | 9.15E-01 | 6.01E-01 | 8.86E-01 | 1.25E-01 | 7.98E-01 |
| TAG58:8 | 8.76E-02 | 7.77E-01 | 4.67E-01 | 8.79E-01 | 1.98E-01 | 7.98E-01 | 6.81E-01 | 8.86E-01 | 8.13E-01 | 9.07E-01 | 3.59E-01 | 8.51E-01 | **4.30E-03** | 6.51E-01 | **1.59E-02** | 7.42E-01 |
| TAG58:9 | 5.57E-01 | 8.86E-01 | 6.98E-01 | 8.87E-01 | 6.57E-01 | 8.86E-01 | 1.08E-01 | 7.88E-01 | 6.91E-01 | 8.86E-01 | 7.15E-01 | 8.87E-01 | **9.42E-03** | 7.42E-01 | **1.87E-02** | 7.42E-01 |
| TAG58:10 | **3.85E-02** | 7.52E-01 | 7.91E-01 | 9.04E-01 | 7.88E-01 | 9.03E-01 | 2.31E-01 | 8.12E-01 | 3.66E-01 | 8.51E-01 | 5.01E-01 | 8.86E-01 | 5.73E-02 | 7.52E-01 | 4.08E-01 | 8.60E-01 |

Linear regressions were performed between the CAD-associated locus (with the CAD-associated allele coded) and the lipid species after log transformation adjusting for age and sex. Q-values were calculated using the QVALUE software. Chol, cholesterylester; DAG, diacylglyceride; LPC, lysophosphatidylcholine; PC, phosphatidyl-choline; PC-O, phosphatidylcholine ether; PE, phosphatidylethanolamine; PE-O, phosphatidylethanolamine ether; SM, sphingomyelin; TAG, triacylglyceride.
